# Methyl-SNP-seq reveals dual readouts of methylome and variome at molecule resolution while enabling target enrichment

**DOI:** 10.1101/gr.277080.122

**Published:** 2022

**Authors:** Bo Yan, Duan Wang, Romualdas Vaisvila, Zhiyi Sun, Laurence Ettwiller

**Affiliations:** 1 New England Biolabs, Incorporated, Ipswich, Massachusetts 01938, USA;; 2 SLC Management, Wellesley Hills, Massachusetts 02481, USA

## Abstract

Covalent modifications of genomic DNA are crucial for most organisms to survive. Amplicon-based high-throughput sequencing technologies erase all DNA modifications to retain only sequence information for the four canonical nucleobases, necessitating specialized technologies for ascertaining epigenetic information. To also capture base modification information, we developed Methyl-SNP-seq, a technology that takes advantage of the complementarity of the double helix to extract the methylation and original sequence information from a single DNA molecule. More specifically, Methyl-SNP-seq uses bisulfite conversion of one of the strands to identify cytosine methylation while retaining the original four-bases sequence information on the other strand. As both strands are locked together to link the dual readouts on a single paired-end read, Methyl-SNP-seq allows detecting the methylation status of any DNA even without a reference genome. Because one of the strands retains the original four nucleotide composition, Methyl-SNP-seq can also be used in conjunction with standard sequence-specific probes for targeted enrichment and amplification. We show the usefulness of this technology in a broad spectrum of applications ranging from allele-specific methylation analysis in humans to identification of methyltransferase specificity in complex bacterial communities.

The covalent modification of cytosine by a methyl group leads to the formation of 5-methylcytosine (5mC), a key epigenetic modification of genomic DNA that occurs in a large number of organisms and represents so far the best-characterized form of DNA modification. In mammals, patterns of methylation are established early during embryogenesis and include X-Chromosome inactivation, imprinting, and the repression of repeats and transposable elements (Greenberg and Bourc'his 2019). Global or regional changes of DNA methylation are among the earliest events known to occur in cancer (Baylin and Jones 2016). Identifying methylation profiles in humans is a key step in studying disease processes and is increasingly used for diagnostic purposes.

In prokaryotes, the vast majority of genomes contain methylated nucleotides (Blow et al. 2016). Contrary to eukaryotes in which the methylation sites are variable and subject to epigenetic states, bacterial methylations tend to be constitutively present at specific sites across the genome. These sites are defined by the methyltransferase specificity and, in the case of restriction modification (RM) systems, tend to be fully methylated to avoid cuts by the cognate restriction enzyme. Current high-throughput techniques for the identification of 5mC are performed by converting cytosine (C) to uracil (which is read as T during sequencing) leaving 5mC intact. This conversion is performed using chemical treatment (bisulfite) or enzymatic treatment (EM-seq). In both methods, this conversion must be complete, leading to the loss of complementarity and separation of the two strands with thymine (T) being either a genuine T or the product of amplification after the deamination of C. Consequently, separate experiments are needed to obtain accurate sequence variants and DNA modification information. A number of hairpin bisulfite sequencing techniques have been developed that lock Watson and Crick strands together by a hairpin adapter followed by bisulfite treatment and PCR amplification or library preparation (Laird et al. 2004; Liang et al. 2021; Füllgrabe et al. 2022; Kyriakopoulos et al. 2022). They use paired-end sequencing to provide both complementary converted sequences that can be deconvoluted to the original four-base sequence in subsequent analytical steps. Adding hairpin adapters before conversion is addressing some of the important shortcomings of conversion-based sequencing, such as the simultaneous readout of variations with epigenetics and the identification of hemimethylation (Burden et al. 2005; Kyriakopoulos et al. 2022). Nonetheless, because both strands are subjected to conversion, neither of the strands retains the original sequence information and thus, cannot be used in conjunction with conventional probe-based enrichment and hybridization techniques.

To circumvent these limitations, we developed Methyl-SNP-seq. Similar to the hairpin adapter method previously published (Liang et al. 2021), we lock the forward and reverse strands together. The innovation is on our new design that permits a dual direct readout of the sequence and the methylation on the same paired-end read (see Fig. 1 for principle). So far, this dual readout was only possible with single-molecule sequencing platforms such as Oxford Nanopore Technologies (Rand et al. 2017; Simpson et al. 2017) and Pacific Biosciences (PacBio) (Clark et al. 2013). Although single-molecule sequencing requires the original DNA molecule to be sequenced to conserve the methylation information, Methyl-SNP-seq can be combined with DNA amplification using a high-throughput Illumina sequencer. Furthermore, the dual readout can be used both at the experimental and analytical steps. Experimentally, the original sequence can be used to enrich targeted regions using conventional four-nucleotide probes matching the original sequence instead of bisulfite-converted probes that are specific to methylation. Subsequently, the methylation and variation can be phased together at molecule resolution while the redundancy of the complementary strands increases the accuracy of variant detection. In addition, methylation motifs can also be directly detected from the sequencing reads without a reference genome.

**Figure 1.**
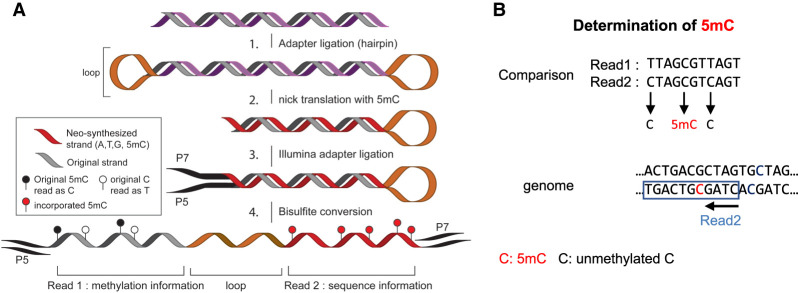
Overview of Methyl-SNP-seq. (*A*) Experimental workflow of Methyl-SNP-seq: (1) The genomic DNA is fragmented to 300–400 bp fragments. (2) Hairpin adapters are ligated at both ends of the fragmented DNA, forming a dumbbell-shaped DNA. Next, nicks at both opposite ends of the adapters are introduced and using nick translation, a copy of the original strand is synthesized, replacing CTP as a source of nucleotide with m5CTP instead. This nick translation step breaks the dumbbell-shaped DNA somewhere within the fragment. (3) Methylated Illumina Y-shaped adapters are ligated. (4) Bisulfite conversion opens the DNA structure revealing a single-stranded DNA molecule that can be amplified using the Illumina adapters. Sequencing requires paired-end reads to obtain both the methylation and the genomic sequence information. For more details on the experimental procedure, see Supplemental Figure 1A and Supplemental Protocol. (*B*) Deconvolution procedure: for more details on the bioinformatics analysis, see Supplemental Figure 1B.

We applied Methyl-SNP-seq on a variety of biological models: First using human genomic DNA, we assessed whether the joint identification of SNPs and methylation is comparable with commonly used techniques. We also took advantage of the dual readout to question whether allele-specific methylation (ASM) can be identified. We next applied Methyl-SNP-seq in conjunction with exome capture and compared the resulting data to standard DNA-seq based exome sequencing. Finally, we applied Methyl-SNP-seq on a bacterium and a synthetic microbial community to identify methylation sites and evaluate whether a reference genome can be de novo–derived from the Methyl-SNP-seq reads.

## Results

### Principle of Methyl-SNP-seq

To capture the original four nucleotide sequence information as well as cytosine modification, Methyl-SNP-seq is taking advantage of the double-stranded nature of DNA to duplicate the sequence information into a linked copy of the original strand. Importantly, this copy is amended to be resistant to bisulfite conversion by replacing all the cytosines with deamination-resistant modified nucleotides. Thus, the copied strand conserves its original four nucleotide content while the original strand undergoes deamination at unmethylated cytosines. Both strands are connected as one molecule and are sequenced using Illumina paired-end sequencing, resulting in one read containing the sequence information while the other paired read contains the methylation information (Fig. 1A; Supplemental Fig. 1A).

To achieve this, a hairpin adapter is ligated to both ends of the fragmented double-stranded DNA, forming a dumbbell-shaped DNA molecule. Next, a nick at both opposite ends of the adapters is introduced, and a copy of the original strand is synthesized via nick translation while the other strand remains unchanged. Nick translation is performed in the presence of 5mdCTP as substrate and thus, the newly synthesized strand is resistant to bisulfite conversion. The nick translation step breaks the dumbbell-shaped DNA somewhere within the fragment, creating two molecules each containing a neo-synthesized strand and the original strand. Methylated Illumina Y-shaped adapters are ligated before bisulfite conversion. Bisulfite conversion deaminates unmodified cytosines to uracils on the original strand and opens the closed DNA structure, revealing a single-stranded DNA molecule that can be amplified using the Illumina adapters. Sequencing requires paired-end reads to obtain both the methylation and the genomic sequence information. We designed the protocol so that the Read1 of the paired-end read pair provides the cytosine methylation information conveyed by bisulfite conversion while the corresponding Read2 provides the genome sequence. To combine both information together, we developed a deconvolution algorithm (Methods; Fig. 1B; Supplemental Fig. 1B) that compares Read1 with Read2 considering the conversion and complementary nature of the paired-end reads. This step, called the read deconvolution step, accurately identifies each cytosine and its methylation status. More specifically, a T in Read1 pairing with a C in Read2 corresponds to an unmethylated C, whereas a C in Read1 pairing with a C in Read2 corresponds to a methylated C (Fig. 1B) while all remaining pairs should match.

A typical Methyl-SNP-seq experiment yields about 85%–90% of the reads being deconvoluted. Within the deconvoluted reads, around 98%–99% of the positions show either a direct agreement between pairs or a profile consistent with cytosine conversion. The remaining 1%–2% of bases that disagreed may be resulting from damages caused by the bisulfite reaction or errors generated during nick translation, PCR amplification, or sequencing. In this case, we cannot differentiate the correct base. Accordingly, we use the Read1 base as the deconvoluted base but adjust the Phred quality score to mark this disagreement as a potential error. Adjustment of the Phred quality scores in the case of a pair disagreement depends on whether a reference genome is available or not. If a reference genome is available (reference-dependent read deconvolution), base calibration is performed using Bayesian statistics, which considers the corresponding nucleotide on the reference genome, the substitution type, and the sequencing cycle. Thus, the adjusted Phred quality score reflects the Bayesian probability that the base recorded on Read1 is true. If a reference genome is unavailable (reference-free read deconvolution), the Phred quality score, in case of pair disagreement, is assigned to 0.

The deconvolution step results in a FASTQ file that contains deconvoluted reads with adjusted Phred quality scores and, for each cytosine, its methylation status in a methylation report file (Supplemental Fig. 1B). The pipeline for processing and deconvoluting the linked paired-end reads is freely available in GitHub (Methods) and Supplemental Code. The outputs of the deconvolution pipeline are in a standard format compatible with existing algorithms designed for genome assembly, genetic variant calling (e.g., GATK [McKenna et al. 2010]), and methylation quantification (e.g., Bismark [Krueger and Andrews 2011]). The ability to distinguish between a methylated and unmethylated cytosine directly on the unmapped read while simultaneously obtaining the original genomic sequences is the key strength of this technology.

### Application of Methyl-SNP-seq to whole-genome sequencing of human GM12878 genomic DNA

As proof of concept, we tested Methyl-SNP-seq using gDNA from the widely studied human cell line GM12878 (lymphoblastoid cell line) for which a large number of sequencing and methylation data sets are publicly available. Unmethylated lambda DNA was spiked into the human gDNA to monitor the bisulfite conversion efficiency. Methyl-SNP-seq libraries were performed in duplicate using the same source of starting material to monitor the reproducibility of the method (Methods). Whole-genome sequencing was performed using Illumina NovaSeq resulting in an average of 1.5 billion 100 bp paired-end reads per replicate. During the deconvolution step of Methyl-SNP-seq, an average of 84% of reads were successfully deconvoluted and more than 95% of the deconvoluted reads were mapped to the reference human genome using Bowtie 2 (Langmead and Salzberg 2012). To obtain a set of high-confidence genetic variants and accurate methylation quantification, we applied stringent data filters to remove multiple mapping reads, PCR duplicates, and reads indicating incomplete bisulfite conversion. About 64% of mapped reads remained after applying these filters. Supplemental Figure 1B shows the data analysis workflow used for this experiment. Both replicates show similar QC and alignment metrics (Supplemental Table 1) indicating that the Methyl-SNP-seq protocol is reproducible.

#### Methyl-SNP-seq accurately detects genetic variation

We first assessed the ability of Methyl-SNP-seq to detect genetic variations in the human GM12878 cell line. To increase coverage, filtered reads from the two replicates were combined for variant calling. Genetic variants were identified using the GATK pipeline (McKenna et al. 2010) following the recommended best practice workflow. The resulting variants were benchmarked against the variants obtained using the NA12878 whole-genome sequencing data set (WGS, performed by the JIMB NIST project). The number of true-positive, false-positive, and false-negative variants found using Methyl-SNP-seq were derived from the comparison between the two data sets. We first confirmed that the reference-dependent read deconvolution increases the number of true-positive SNPs and reduces the number of false-positive SNPs compared with reference-free read deconvolution (Supplemental Fig. 2A).

**Figure 2.**
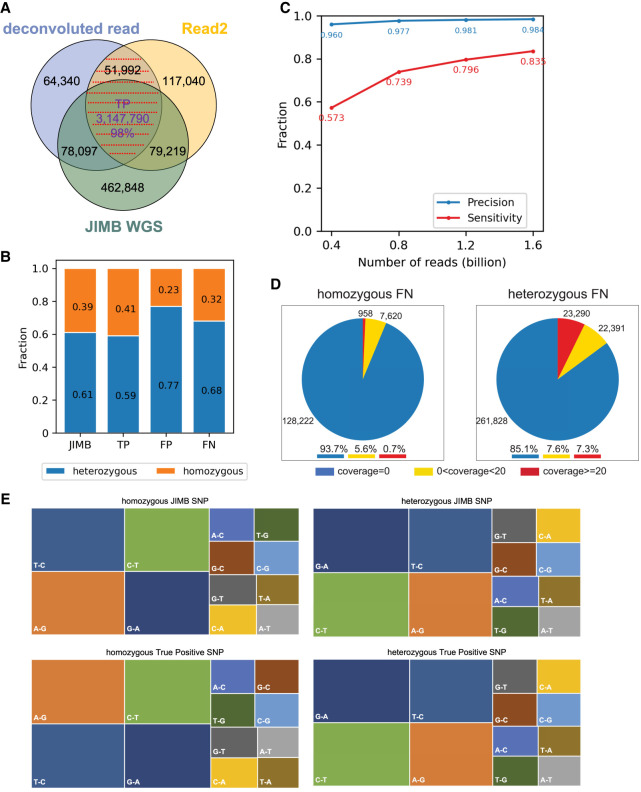
SNP identification. (*A*) Comparison of SNPs identified using Methyl-SNP-seq deconvoluted read and Read2 with those using JIMB whole-genome sequencing data. Common SNPs, which were identified by both deconvoluted read and Read2 and marked by red dashed lines, are referred to as Methyl-SNP-seq defined SNPs. (*B*) Fraction of heterozygous and homozygous Methyl-SNP-seq defined SNPs. (*C*) Precision and sensitivity of SNP identification using different numbers of Methyl-SNP-seq reads. Precision = TP/(TP + FP). Sensitivity = TP/(TP + FN) with TP: True positive. FP: False positive. FN: False negative. (*D*) Distribution of the genome coverage of the false-negative SNP sites. (*E*) Characterization of the JIMB and true-positive Methyl-SNP-seq defined SNPs (i.e., T-C means in the VCF file REF = T while ALT = C). The JIMB whole-genome sequencing of NA12878 was used as a benchmark for comparison.

Because both deconvoluted read and Read2 represent the original genome sequence, we can call genetic variants using either one. Overall, variants found using either the deconvoluted read or Read2 show a high level of agreement: 95% of SNPs found with the deconvoluted read being identical to those found with Read2 (Fig. 2A). According to the experimental design, as expected the deconvoluted read and Read2 had different types of false-positive errors (Supplemental Fig. 2B). Consequently, by using the common set of variants defined by both deconvoluted read and Read2, we could correct the variant calling error and improve the accuracy (Supplemental Fig. 2C). Therefore, we chose the common variants between deconvoluted read and Read2 as the Methyl-SNP-seq defined genetic variants.

Using this set of common variants, we found 1,296,699 and 1,903,083 homozygous and heterozygous SNPs, respectively (see Data access). A total of 98% of the SNPs identified using Methyl-SNP-seq were confirmed by JIMB WGS with a better agreement for homozygous SNPs (accounting for one-fifth of the total false-positive SNPs) compared with heterozygous SNPs (accounting for 80% of the total false-positive SNPs) (Fig. 2A,B). Our method also shows high accuracy for indels, with 94% agreement with WGS. These levels of agreement are comparable to those typically observed between standard WGS (Zook et al. 2014).

We performed standard quality controls for variant calling (Picard CollectVariantCallingMetrics) and found that both the Methyl-SNP-seq and the WGS data sets displayed comparable metrics for both SNPs and indels (Supplemental Table 2). More specifically, the profiles of true-positive SNPs substitution observed in Methyl-SNP-seq are similar to the ones obtained by WGS (Fig. 2E) and the ratio of transition (Ti) to transversion (Tv) mutations is around 2.06 for both data sets demonstrating that both sets are unlikely to have a bias affecting the transition transversion ratio. As expected, we have less accuracy in detecting the C-T (REF-ALT in VCF), T-C, G-A, and A-G type SNPs, which, combined, account for most of the errors (Supplemental Fig. 3).

To assess the sensitivity of Methyl-SNP-seq in identifying variants, we downsampled the data set to various fractions of total reads. At equivalent coverage, we detected more than 80% of the WGS SNPs. This number drops to 60% when using only 25% of reads (Fig. 2C). We noted that the lack of read coverage was the major cause of false-negative SNPs from Methyl-SNP-seq (Fig. 2D). Although having the same number of reads, the number of total bases was fewer in deconvoluted reads (109 billion) compared with WGS (160 billion) owing to the shorter read length after trimming the hairpin adapter. Importantly, reducing the number of reads did not affect the accuracy of variant detection (Fig. 2C).

#### Methyl-SNP-seq accurately detects and quantifies cytosine methylation at base resolution

We next evaluated the performance of Methyl-SNP-seq in identifying and quantifying cytosine methylation. The methylation status of individual cytosine was determined in the read deconvolution step and was added to the mapped BAM file so that the base-resolution methylation information can be calculated using conventional methylation calling tools such as Bismark (Krueger and Andrews 2011; Methods). We benchmarked our method's performance against two reference data sets generated by the standard whole-genome bisulfite sequencing (WGBS) method (The ENCODE Project Consortium 2012; Davis et al. 2018) and Nanopore sequencing (Jain et al. 2018). Using the unmethylated lambda spiked-in control, we estimated the bisulfite conversion rate of Methyl-SNP-seq to be 97.5%. Overall CpG methylation is at 45.3% for both replicates in line with the two ENCODE data sets analyzed showing 45.5% and 50.1% overall CpG methylation, respectively. The GC bias of Methyl-SNP-seq follows closely the known GC bias observed for bisulfite sequencing (Olova et al. 2018) with a preferential sequencing of AT-rich genomic regions. Both replicates show comparable results (Supplemental Table 3).

**Figure 3.**
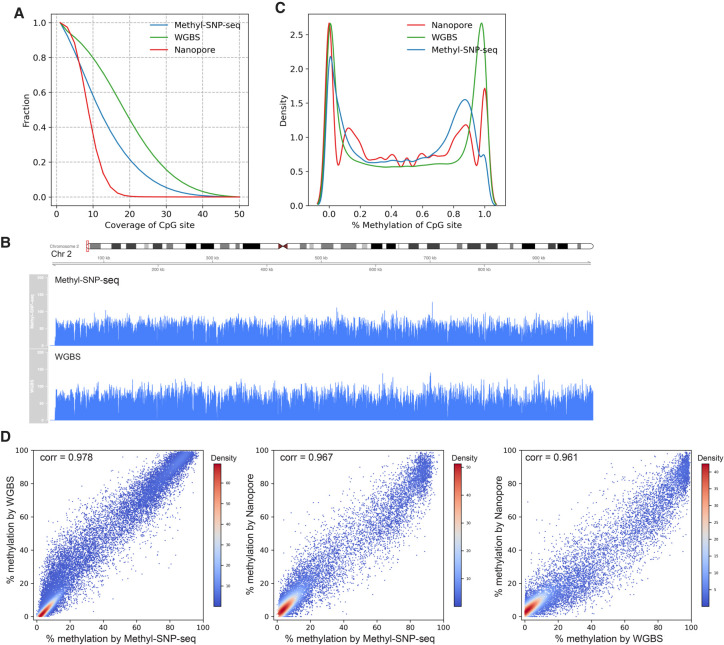
Methylome. (*A*) Distribution of coverage on CpG sites for Methyl-SNP-seq (blue), whole-genome bisulfite sequencing (WGBS) (green), and Nanopore (red). (*B*) Read coverage on part of the human Chr 2 for Methyl-SNP-seq (*top*) and WGBS (*bottom*). (*C*) Distribution (kde plot) of % methylation on CpG sites having coverage ≥ 5. (*D*) Pairwise comparison of the methylation level of CpG islands measured by Methyl-SNP-seq, whole-genome bisulfite sequencing from ENCODE (WGBS), and Nanopore sequencing. Each dot represents the percentage methylation at CpG island. Only CpG islands having coverage ≥ 50 were used for correlation calculation. A total of 27,050; 27,313; and 16,071 CpG islands were detected by Methyl-SNP-seq, WGBS, and Nanopore sequencing, respectively.

With 1.6 billion reads from the two replicates combined, we acquired 54 million CpG sites, for which 45 million had at least 5× coverage (see Data access). These numbers are comparable with that of the WGBS method downsampled to a similar number of reads (53 million sites with ≥5× coverage) (Fig. 3A,B). The genome-wide methylation level of CpG sites identified by Methyl-SNP-seq displays a bimodal distribution similar to those of the WGBS and Nanopore data sets (Fig. 3C) with a distribution that better resembles the Nanopore data set. This result agrees with the observation that bisulfite sequencing overestimates the global methylation (Ji et al. 2014; Jain et al. 2018; Olova et al. 2018).

Methylation patterns of CpG islands have been shown to affect gene expression and are linked to disease phenotypes (Robertson 2005). Therefore, we compared the methylation level of the known CpG islands obtained using the three methods. Methyl-SNP-seq is highly correlated with both the ENCODE WGBS (Pearson's correlation = 0.98) and Nanopore (Pearson's correlation = 0.97) data sets (Fig. 3D), indicating that Methyl-SNP-seq is highly accurate for cytosine methylation quantification.

#### Allele-specific methylation using Methyl-SNP-seq

Attempts to infer SNPs from WGBS have been previously published (Liu et al. 2012) but require high genome coverage (e.g., >30× coverage required by Bis-SNP) to assess independently paired-end reads. This is because the identification of transition SNP such as C/T, G/A, A/G, and T/C are confounded by the deamination step (Liu et al. 2012). In contrast, our method can confidently distinguish cytosine methylation from original transition SNPs along with other SNP types. Indeed, by using the redundancy of the double-stranded DNA to read methylation and sequence from the same original DNA molecule, our method identifies both the methylation state and variants at the single-molecule level. This allows phasing of the methylation state with heterogeneous SNPs directly on the read, enabling the identification of differentially methylated genomic regions (DMRs) that are allele specific (ASDMR).

**Figure 4.**
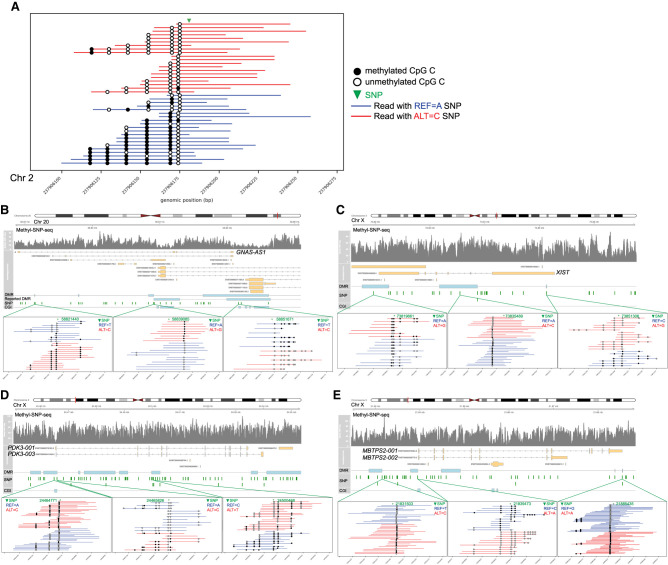
Differentially methylated genomic regions (DMRs). (*A*) Chr 2: A known example of allele-specific methylation. The reads having different alleles of the arrow-pointed heterozygous SNP site are labeled with blue (REF SNP) and red (ALT SNP). SNP: rs11686156. (*B*) Chr 20: 10-kb upstream of *GNAS-AS1* gene and overlapping reported DMRs (Fang et al. 2012). (*C*) Chr X: *XIST*. (*D*) Chr X: *PDK3*. (*E*) *MBTPS2*. Only Cs in the CpG context are shown. Coordinates on the *x*-axis correspond to positions on respective chromosomes (GRCh38 assembly).

Using the whole-genome Methyl-SNP-seq experiment performed on human NA12878 described above, we identified a total of 34,909 ASDMRs genome-wide. Figure 4A shows an example of a known ASDMR (Kaplow et al. 2015; Suzuki et al. 2018) containing the heterozygous SNP rs11686156 on Chr 2. Among all the identified ASDMRs, 47% have SNP directly affecting CpG sites. This result is consistent with a previous study (Shoemaker et al. 2010), which reported that 38%–88% of ASM regions are solely caused by the presence of SNPs at CpG dinucleotides and indicates variation at CpG sites is a dominating factor for ASDMR. In this case, SNP not only disrupts the methylation pattern of the affected CpG site but also affects the methylation pattern of the neighboring regions. Therefore, CpG-SNPs are very important for DMR studies because they may play a role in the establishment of certain types of DMRs such as ASDMRs.

Allele-specific methylation is also often associated with gene imprinting. Using a set of ASDMRs that are reported to be associated with known imprinted gene clusters in the human genome as a reference (Fang et al. 2012), we were able to identify 24 ASDMRs at or near the reported imprinting control DMRs for 15 out of the 30 imprinted gene clusters (Supplemental Table 4). For example, we detected 2 ASDMRs overlapping with the known imprinted cluster of the *GNAS-AS1* gene (Fig. 4B). These two ASDMRs span a 17.8-kb region and include 670 CpG pairs. When examining the methylation level of all the CpGs within this region, we saw that most of the CpG sites have an average methylation level close to 50% whereas CpG sites in the flanking regions have elevated methylation levels, suggesting this entire 17.8-kb region is likely an imprinted DMR (Supplemental Fig. 4).

Allele-specific methylation (ASM) is also known to be associated with X-Chromosome inactivation in female cells via regulating the X-inactive specific transcript (*XIST*) gene (Wutz 2011; Fang et al. 2012). Accordingly, our method detected several ASMs near the *XIST* gene in the human lymphocyte cell GM12878 (female) (Fig. 4C). In addition, we also detected ASMs in the promoter regions of genes that are known to be subject to X-Chromosome inactivation (XCI) (Sharp et al. 2011; Cotton et al. 2015) such as *PDK3* and *MBTPS2* (Fig. 4D,E).

A previous study (Kaplow et al. 2015) found that genomic regions with chromatin states consistent with active transcription and active enhancers were enriched for CpGs with mQTLs (ASM), suggesting that some of these ASMs may affect transcription or enhancer activity. In our study, we found that CpGs that are associated with ASDMRs are significantly enriched, compared with random CpG regions, in enhancers, which include both active and primed enhancers and are marked by histone H3K4me1 modification in the absence of histone H3K4me3 modification (χ^2^ = 98.3, df = 1, *P*-value < 1 × 10^−9^, fold change = 1.5) (Supplemental Table 5 in Supplemental Material). However, ASDMR CpGs are not enriched in active enhancers identified by H3K27ac modification (fold change = 0.9). In addition, ASDMR CpGs are significantly depleted in the promoter regions marked with histone H3K4me3 modification (χ^2^ = 120.1, df = 1, *P*-value < 1 × 10^−9^, fold change = 0.7). ASDMR CpGs are also enriched in the genomic regions with repressive histone mark H3K9me3 (χ^2^ = 29.1, df = 1, *P*-value = 6.8 × 10^−8^, fold change = 1.4). This histone mark is associated with heterochromatin and frequently coexists with DNA methylation. H3K9me3 is also reported to play a role in establishing imprinted X-Chromosome inactivation in mice (Fukuda et al. 2014).

### Methyl-SNP-seq can be performed in conjunction with the conventional probe-based target enrichment

Although providing a comprehensive view of the human genome, whole-genome sequencing remains cost-prohibitive for analyzing a large number of clinical samples. In contrast, targeted sequencing focusing on specific regions of interest is more widely and commonly used. In particular, targeted bisulfite sequencing is designed to measure site-specific DNA methylation changes. Accordingly, it normally requires the design of specific bait probes capturing the bisulfite-converted regions. Unlike the conventional targeted bisulfite sequencing, the Methyl-SNP-seq method contains the original genome sequence (Fig. 1) that can hybridize with the standard bait probes. Thus, in theory, Methyl-SNP-seq can be easily adapted to the conventional targeted enrichment method with any standard probe set.

**Figure 5.**
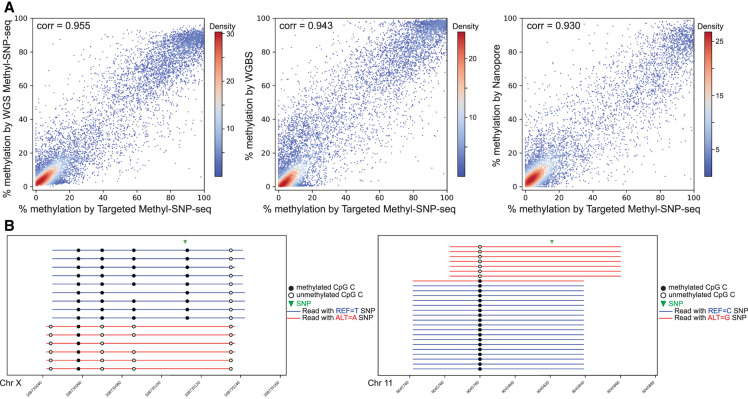
Targeted Methyl-SNP-seq. (*A*) Pairwise comparison of the methylation level of CpG islands measured by targeted Methyl-SNP-seq, whole-genome Methyl-SNP-seq (WGS Methyl-SNP-seq), whole-genome bisulfite sequencing by ENCODE (WGBS), and Nanopore sequencing. Only CpG islands having coverage ≥ 50 were used for correlation calculation. (*B*) Two allele-specific methylations (ASMs) depicted by targeted Methyl-SNP-seq. Only Cs in CpG context are shown. Coordinates on the *x*-axis correspond to positions on Chr X (*left*) and Chr 11 (*right*) (GRCh38).

To show the applicability of Methyl-SNP-seq for target enrichment, we tested Methyl-SNP-seq combined with the Twist human comprehensive exome panel. The targeted Methyl-SNP-seq had a high mapping efficiency with 96% of the reads mapping to the human genome. The bisulfite conversion rate is 97% in CHG and CHH contexts. Targeted Methyl-SNP-seq also showed comparable target enrichment capability (Supplemental Table 6) compared with the standard exome targeted capture with the exception that it had lower coverage of AT-rich regions (AT_DROPOUT = 9). We also applied stringent filters to remove PCR duplicates, etc., consequently having about 11 million deconvoluted reads from two replicates. Although the Twist exome panel is designed to capture the gene body rather than the promoter regions, we still found 11,783 CpG islands captured with coverage above 50. Like the whole-genome sequencing, the methylation quantification of these CpG islands was consistent with the WGBS and Nanopore result (Fig. 5A). As for the variant detection, using the same probe panel at equivalent read depth (about 10 million reads used for variant calling), the precision of targeted Methyl-SNP-seq sequencing (precision = 0.8) was lower than the standard targeted sequencing (precision = 0.9) (Zhou et al. 2021). As for Methyl-SNP-seq, targeted Methyl-SNP-seq can also be used for the identification of ASM (Fig. 5B).

### Reference-free identification of m5C in bacteria using Methyl-SNP-seq

Another application of Methyl-SNP-seq is on the identification of methylation in organisms for which a reference genome or assembly is missing. This is often the case for environmental samples and microbiomes. In these cases, conversion-based methods to identify methylation (e.g., bisulfite sequencing) cannot be used because these methods rely on differentiating between a genuine T and a C-to-T conversion using a reference genome. The Methyl-SNP-seq method, on the other hand, identifies cytosine methylation directly on the paired-end reads in a reference-independent manner. Additionally, it reports the methylation status of individual cytosine sites with sequence context information at single-base resolution and at single-molecule level, which is most suitable for methylation motif studies. Furthermore, our Methyl-SNP-seq method also reports the original genomic sequences that can be used for genome assemblies of a single organism or a mixed population.

**Figure 6.**
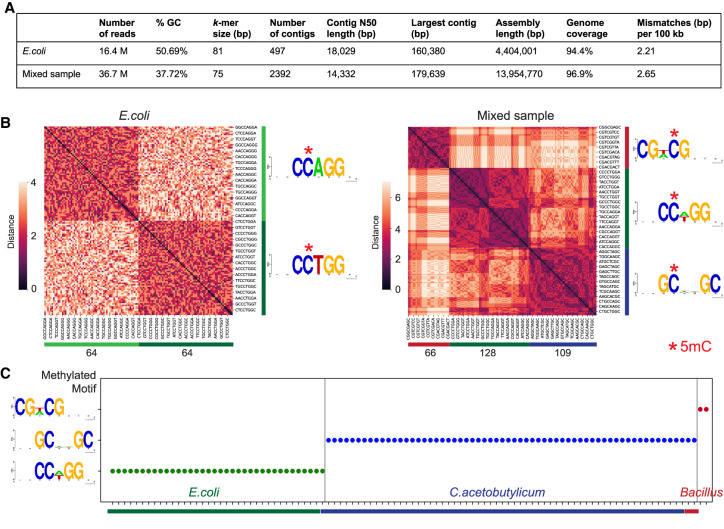
Methyl-SNP-seq applied to bacteria. (*A*) Summary of genome assembly using deconvoluted reads. Genome coverage was measured against *E. coli* or *E. coli* + *C. acetobutylicum* reference genome using QUAST. (*B*) Hierarchy clustering of significantly enriched 8-mers and the corresponding motif logo (generated by WebLogo [Crooks et al. 2004]) for *E. coli* and mixed sample (*E. coli* + *C. acetobutylicum*). The number at the *bottom* represents the number of enriched *k*-mers in each cluster. (*C*) Identified methyltransferase motif for the top 100 largest assembled contigs. Each dot represents a contig.

To show the effectiveness of Methyl-SNP-seq for these applications, we performed Methyl-SNP-seq using the genomic DNA of an isolated strain of *Escherichia coli* K12. We first investigated whether we could assemble the deconvoluted reads into a reliable reference genome. Using the Velvet assembler (Zerbino 2010), we obtained a good assembly from the *E. coli* data (16 million deconvoluted reads) with high genome coverage (94% of the genome covered) and high sequence identity (2.21 mismatches per 100 kbp) (Fig. 6A), which was comparable to the performance of the assembler for single-end short read assembly using standard DNA-seq.

We next determined the methyltransferase specificity of the bacteria directly on the deconvoluted reads without mapping to a reference genome. To achieve this, we randomly selected 0.3 million deconvoluted reads and counted the number of occurrences of all the 8 bp *k*-mers (8-mers) having methylated or unmethylated cytosine from these reads. We applied a binomial model with Bonferroni adjustments to identify the 8-bp sequences having significantly higher methylation levels. These sequences were further grouped using hierarchical clustering to uncover the consensus methylated motif indicative of methyltransferase specificity(ies). Two context clusters were found from 128 significantly enriched 8-mers. CCAGG and CCTGG can be further combined into CCWGG (with W = A or T), revealing the correct specificity for the *E. coli* Dcm methyltransferase (Fig. 6B; Marinus and Morris 1973; May and Hattman 1975).

We also performed Methyl-SNP-seq on a mixed sample consisting of genomic DNA of two bacterial strains (*E. coli* K12 and *Clostridium acetobutylicum* ABKn8 strains) to mimic a simple mixed bacteria population. Using 0.6 million deconvoluted reads, we found three motifs instead of the two motifs expected for these strains (Fig. 6B). These motifs correspond to CCWGG, GCNNGC, and CGWCG. The first two motifs match the expected methyltransferase specificities of *E. coli* Dcm and *C. acetobutylicum* (Baum et al. 2021), respectively. The third motif, CGWCG, however, was unexpected. To investigate the origin of this methylated motif, we assembled the deconvoluted reads of the mixed sample library and used the 100 longest assembled contigs as reference to determine methylation motifs. All of the 100 contigs contain a single methylated motif, among which 36 and 62 have the CCWGG motif and the GCNNGC motif, consistent with their *E. coli* and *C. acetobutylicum* origins (Fig. 6C). Two out of the 100 contigs have the unexpected CGWCG motif and both contigs have high sequence homology with the genome sequence of a *Bacillus* strain by BLAST search, implying that there was a contamination and most likely a *Bacillus* strain in the mixed sample. We further annotated the assembled contigs, which contain the CGWCG motif using Prokka and identified a single gene with cytosine-specific DNA methyltransferase domain. This suggests the contamination strain is methylated. Additionally, the methylation analysis using EM-seq confirmed the presence of a CGWCG methylation motif in the used *C. acetobutylicum* sample.

In this example, we showed that the Methyl-SNP-seq method can identify all the methylation motifs from a mixed sample in a reference-independent manner, as well as resolve the composition of a mixed population by assembling the deconvoluted sequences and using methylation motif as a species/strain signature and genome binning criteria.

## Discussion

Amplification-based sequencing methods provide only the sequential arrangement of the canonical four bases A, T, C, and G while all modifications, originally present on the DNA, are erased. The information on what base was originally modified is lost during the in vitro DNA synthesis steps that happen during amplification, clustering, and sequencing. To circumvent this limitation and obtain cytosine methylation information, techniques such as bisulfite sequencing convert unmethylated Cs to Ts before subjecting the converted DNA to sequencing. A T output after bisulfite treatment is therefore ambiguous: It corresponds to either a naturally occurring T in the sequence or a deaminated unmodified C and a reference genome is therefore required to distinguish the two possibilities. This ambiguity is the major drawback in bisulfite sequencing and relegates all the techniques that rely on deamination to applications directed for methylation analysis only.

By locking the double strands together, Methyl-SNP-seq takes advantage of the redundant information captured in the complementary strands to obtain both the arrangement of the canonical four bases and the methylation information. The accuracy of the dual readouts of Methyl-SNP-seq is comparable to state-of-the-art techniques for both SNPs and methylation analysis. Because the sequencing power is allocated to a dual readout, the sensitivity for every single readout is reduced to effectively a single-end read instead of a paired-end read. This affects notably the ability to perform assemblies as most of the assemblers have been optimized for paired-end sequencing. With the ability to read longer stretches of sequence, this limitation can be partially overcome. Furthermore, there should be no technical limitations from the manufacturer in adapting the instrument to perform dual paired-end read sequencing using primer targeting the invariable hairpin structure. The dual readout also implies that the sequencing volume to achieve the similar sensitivity obtained by WGBS or DNA-seq needs to be increased. Notably, compared with the published hairpin approaches, Methyl-SNP-seq requires twice the sequencing volume to obtain similar methylation information, a drawback that can be largely compensated by the ability to perform target enrichment, which significantly cuts down on sequencing cost if whole-genome information is not required.

It is also possible to first capture genomic DNA before performing deamination (Lee et al. 2011) using probes designed using the conventional four bases sequences. Nonetheless, this strategy has two main limitations compared with capture-Methyl-SNP-seq: (1) To preserve the methylation information, no DNA amplification before capture is feasible, limiting the range of applications; and (2) once captured, the DNA fragment is single-stranded, preventing the use of hairpin approaches. Consequently, capturing the original DNA fragment first can only be combined with regular bisulfite sequencing.

Compared with the published hairpin approaches, Methyl-SNP-seq is required to neo-synthesize one of the double strands and thus, the methylation status of the CpG pair such as hemimethylation is lost. On the other hand, it is not possible to perform target enrichment using the published hairpin approaches.

Methyl-SNP-seq is a single experiment that is more convenient than performing WGBS and DNA-seq separately. In addition, Methyl-SNP-seq offers important functionalities that are not feasible when performing WGBS or DNA-seq. Notably, Methyl-SNP-seq preserves the base arrangement of one of the double strands by incorporating m5CTP instead of CTP in the neo-synthesized fragment. This is conceptually a significant improvement compared with previously published methods (Laird et al. 2004; Liang et al. 2021; Füllgrabe et al. 2022; Kyriakopoulos et al. 2022) for which both strands are subjected to deamination. In the latter case, the ability to obtain the original sequence can only be performed computationally, by aligning and deconvoluting paired-end reads. In addition, by keeping intact the four-nucleotide-based strand, Methyl-SNP-seq is compatible with conventional probe sets for target enrichment. Indeed, we show similar on-target performance for both conventional DNA-seq and Methyl-SNP-seq exome sequencing. Methyl-SNP-seq exome sequencing shows an SNP detection accuracy lower than conventional exome sequencing. Thus, a higher coverage is likely needed to obtain similar SNP accuracy. Methyl-SNP-seq also has a very high mapping rate with 95% of the deconvoluted reads mapping to the human genome, which is very similar to standard DNA-seq. We hypothesize that this high mapping rate is correcting the inflation of methylation levels that are typically observed in WGBS (Olova et al. 2018).

Retaining the original sequence is also useful for any target-specific amplification such as CRISPR-based targeting, and other sequence-specific technologies. Beyond sequence-specific applications, we show the applicability of Methyl-SNP-seq in directly demonstrating allele-specific methylation at single-molecule resolution. In conjunction with target-specific amplification and sequencing, Methyl-SNP-seq is an ideal technique to validate candidate ASMs derived from methylome-wide association studies.

Beyond human methylomes, Methyl-SNP-seq is a useful technology notably for organisms for which a reference genome is not available such as nonmodel organisms and microbial communities. Notably, the identification of modification directly on the unmapped reads enhanced the ability to bin sequences based on methylation patterns, an important feature for resolving genomes within a complex community (Tourancheau et al. 2021; Wilbanks et al. 2022). The ability to obtain the original genomic sequence allows further functionalities specific to organisms for which a reference genome is unavailable or variations between the studied organism and its reference genome are too high to confidently distinguish methylation from transition SNPs. For example, we show the ability to perform assemblies and overlay methylation on the newly assembled genome.

We have not optimized the Methyl-SNP-seq protocol for low input. For human whole-genome sequencing, we were able to make enough of the Methyl-SNP-seq libraries for Illumina NovaSeq sequencing using 2 µg input. It is possible to start with a lower amount, such as 20 ng, for application on a single bacterial sample based on our test using *E. coli* genomic DNA.

In summary, this study provides a complete assessment of Methyl-SNP-seq in terms of performances and relevancy for diverse applications ranging from human to bacteria. We performed Methyl-SNP-seq on complete genomes, exomes, single organisms, and mixed populations. For each of these applications, we have shown the abilities to perform methylation and variant calling, phasing of both genetic and epigenetic information, identification of methylase specificity, the ability to de novo–assemble genomes, and resolve species composition in case of a mixed population. These broad assessments of the technology together with open-access downstream analysis strategies should streamline the use of Methyl-SNP-seq to gain insights into epigenetic processes for both eukaryotes and prokaryotes.

## Methods

### Preparation of Methyl-SNP-seq library

For human Methyl-SNP-seq sequencing, we used genomic DNA isolated from the GM12878 cell line (NA12878, provided by Coriell Institute) for library preparation. For human whole-genome Methyl-SNP-seq sequencing, we used 4 µg of NA12878 gDNA and the unmethylated lambda DNA as spiked in to monitor the bisulfite conversion efficiency. The genomic DNA was fragmented using 250 bp sonication protocol using a Covaris S2 sonicator. We set up two technical replicates. For human exome targeted Methyl-SNP-seq sequencing, 4 µg of NA12878 gDNA was fragmented using 400 bp or 500 bp sonication protocol.

For bacteria Methyl-SNP-seq sequencing, we used 2 µg of *E. coli* genomic DNA (MG1655 strain) or 2 µg of mixed bacterial DNA (containing 1 µg of *E. coli* MG1655 genomic DNA and 1 µg of *C. acetobutylicum* genomic DNA). The genomic DNA was fragmented using 250 bp sonication protocol.

As shown in Supplemental Figure 1A, the fragmented gDNA was end-repaired and dA-tailed (NEB Ultra II E7546 module), then ligated to a custom hairpin adapter using NEB ligase master mix (NEB, M0367). The incomplete ligation product (fragment having only one or no adapter ligated) was removed using exonuclease (NEB ExoIII M0206 and NEB ExoVII M0379). Two nick sites were created at the uracil positions in the hairpin adapters at both ends after being treated with UDG (NEB, M0280) and EndoVI (NEB, M0304). The nick sites were translated toward 3′ terminus by DNA polymerase I (NEB, M0209) in the presence of dATP, dGTP, dTGP, and 5-methyl-dCTP. The nick translation causes double-stranded DNA break when DNA polymerase I encounters the other nick on the opposite strand. The resulting fragments have one end ligated to a hairpin adapter and a blunt end on the other side. The blunt end was dA-tailed and ligated with Y-shape methylated Illumina adapter (NEB methylated adapter, E7536). The ligated product was bisulfite converted using Abcam Fast Bisulfite conversion kit (Abcam, ab117127). The bisulfite-converted product was amplified using NEBNext Q5U Master Mix (NEB, M0597) (discussion on Library size-distribution in Supplemental Text; Supplemental Fig. 5). The resulting indexed library was used for Illumina sequencing or target enrichment.

To perform targeted sequencing, about 200–300 ng Methyl-SNP-seq indexed library was used in a pool for target enrichment. The whole-human exome regions were enriched from the pooled libraries using the Twist Human Core Exome panel (Twist, 102025) following the manufacturer's instructions. The enriched DNA fragments were further amplified using NEBNext Q5 Master Mix (NEB, M0544) and NEBNext Library Quant Primer Mix (NEB, E7603) for sequencing following the manufacturer's instructions.

The human Methyl-SNP-seq libraries (WGS sequencing and targeted sequencing) were sequenced using an Illumina NovaSeq 6000 sequencer for 100 bp paired-end reads. The bacteria Methyl-SNP-seq libraries (*E. coli* or mixed sample) were sequenced using an Illumina NextSeq 550 sequencer for 150 bp paired-end reads.

The sequence of the custom hairpin adapter (46 bp) is: 5′-(p)CCACGACGACGACGACGAGCGTTAGGCTCGTCGTCGTCGTCGUGGT-3′

A detailed protocol is provided as Supplemental Protocol in Supplemental Material.

### Preparation of EM-seq library

We used 100 ng of *C. acetobutylicum* genomic DNA to prepare an EM-seq library (NEB E7120) as directed by the manufacturer. The library was sequenced using an Illumina NextSeq 550 sequencer for 75 bp paired-end reads.

### Data analysis

- Reference genome and other annotation files:
  GRCh38 human reference genome:
  ftp://ftp.ncbi.nlm.nih.gov/genomes/all/GCA/000/001/405/GCA_000001405.15_GRCh38/seqs_for_alignment_pipelines.ucsc_ids/GCA_000001405.15_GRCh38_no_alt_analysis_set.fna.gz
  Lambda reference genome:
https://www.ncbi.nlm.nih.gov/nuccore/215104
  Known human SNP files used for GATK Base Quality Recalibration:
  ftp://ftp.broadinstitute.org/bundle/hg38/dbsnp_138.hg38.vcf.gz
  ftp://ftp.broadinstitute.org/bundle/hg38/Mills_and_1000G_gold_standard.indels.hg38.vcf.gz
  ftp://ftp.broadinstitute.org/bundle/hg38/1000G_phase1.snps.high_confidence.hg38.vcf.gz
  JIMB NA12878 SNP VCF file used for ASM identification:
  ftp://ftp-trace.ncbi.nlm.nih.gov/giab/ftp/release/NA12878_HG001/NISTv3.3.2/GRCh38/HG001_GRCh38_GIAB_highconf_CG-IllFB-IllGATKHC-Ion-10X-SOLID_CHROM1-X_v.3.3.2_highconf_PGandRTGphasetransfer.vcf.gz
  Human CpG island annotation was generated using the “CpG island track” from UCSC browser.
- Data processing for Methyl-SNP-seq:
  The commands used for data processing are provided in Supplemental Material.

The sequencing reads were trimmed for both the Illumina adapter and the hairpin adapter (https://github.com/elitaone/Methyl-SNP-seq/tree/main/Read_Processing/TrimRead.py) using Trim Galore! version 0.6.4 (https://github.com/FelixKrueger/TrimGalore). For human NA12878 Methyl-SNP-seq sequencing, the bases of the last cycle (cycle 100) for both Read1 and Read2 were further trimmed because of poor quality.

Next, we performed read deconvolution, which determines the base, adjusts the base quality score, and extracts the methylation information by comparing the paired Read1 and Read2. This step generates a FASTQ file containing the deconvoluted reads and a corresponding methylation report. The principle of read deconvolution is explained as follows (Fig. 1B).

#### Reference-free read deconvolution

If the reference genome is not available, for example, Methyl-SNP-seq for *E. coli* and mixed bacteria sample, a reference-free deconvolution was performed using a custom pipeline (https://github.com/elitaone/Methyl-SNP-seq/tree/main/Read_Processing/DeconvolutionConversion_v2.py), including the following steps:

(1) Base determination and methylation extraction. For the same Illumina cycle, if Read1 base is a C and Read2 base is a C, it results in a C in the deconvoluted read and a 5mC in the methylation report; whereas if Read1 base is a T and Read2 base is a C, it results in a C in the deconvoluted read and an unmethylated C in the methylation report.
(2) Base quality score adjustment. For the mismatching positions that Read1 bases are different from Read2 bases except for the Read1-T Read2-C case, Read1 bases are used but the sequencing quality scores are adjusted to 0 in the deconvoluted reads.

#### Reference-dependent read deconvolution (Supplemental Fig. 1B)

If the reference genome is available, for example, Methyl-SNP-seq for human NA12878, a reference-dependent deconvolution was performed to increase the performance (https://github.com/elitaone/Methyl-SNP-seq/tree/main/Read_Processing/Deconvolution WithCalibration). The step (1) base determination and methylation extraction is the same as the reference-free read deconvolution. But reference-dependent read deconvolution uses a statistical model for the base quality score adjustment in step (2) as shown below.

(2) Base quality score adjustment. For the mismatching positions, by comparing to the reference genome, a Bayesian probability is calculated, which reflects the likelihood of being able to trust the Read1 base. Therefore, Read1 bases are used but the sequencing quality scores are adjusted based on the Bayesian probability in the deconvoluted reads.

In a typical Methyl-SNP-seq experiment, about 10%–15% of reads are undeconvolutable. Data suggest that the partial exonuclease activity to eliminate unligated/partially ligated fragments is the main reason for undeconvoluted reads in our sequencing result. Incomplete digestion by the exonuclease results in regions of single-stranded DNA that are subsequently filled in during the end-repair step resulting in double-stranded DNA molecules that can be ligated and amplified. After sequencing, these fragments represent conventional paired-end bisulfite reads mapping to flanking regions of the genome with the exception that the fill-in regions are resistant to bisulfite conversion. This scenario is supported by the fact that (1) a notable fraction of the undeconvoluted reads map to the genome close to their pair and (2) an optimized exonuclease treatment decreased the percentage of undeconvoluted reads.

#### Alignment and data filtering for human NA12878 Methyl-SNP-seq (Supplemental Fig. 1B)

For human NA12878 Methyl-SNP-seq, the deconvoluted reads were aligned to the GRCh38 human reference genome using Bowtie 2 (version 2.3.0) default parameter for single-end mapping with the addition of read group identifier defined by ‐‐ rg-id and ‐‐rg. These identifiers (including the information for the sequencing platform, flow cell, lane, barcode, and sample) were necessary for Base Quality Score Recalibration by GATK for variant calling.

To achieve high accuracy, the following steps were taken to filter the aligned data before variant calling and methylation status determination:

(1) removal of multiple mapping using a custom script (https://github.com/elitaone/Methyl-SNP-seq/tree/main/Read_Processing/MarkUniread.py). Here, for Bowtie 2 single-end mapping, the unique mapping is defined as the read having only AS tag or AS score != XS score (Bowtie 2 AS: best alignment score, XS: second-best alignment score);
(2) removal of PCR duplicates using a custom script (https://github.com/elitaone/Methyl-SNP-seq/tree/main/Read_Processing/MarkDup.py). Here, for Bowtie 2 single-end mapping, the PCR duplicates are identified as reads aligned to the same position as well as having the same sequence;
(3) addition of an XM tag reflecting the methylation status. Based on the methylation report generated in the read deconvolution step, an XM tag is added to each mapped read in the sam file using a custom script (https://github.com/elitaone/Methyl-SNP-seq/tree/main/Read_Processing/AddXMtag.py). The XM tag is defined by Bismark to mark methylation call string and used to extract methylation status;
(4) removal of reads having incomplete bisulfite conversion using Bismark (version 0.22.3) filter_non_conversion.

The resulting filtered deconvoluted reads from two replicates were combined to be used for variant calling and methylation determination. There were 1.6 billion and 11 million filtered deconvoluted reads for human WGS and exome-targeted Methyl-SNP-seq, respectively.

#### Data processing for human NA12878 whole-genome sequencing

Whole-genome sequencing of human NA12878 generated by JIMB NIST Genome in a Bottle (Zook et al. 2016) (JIMB WGS HG001) was used as a benchmark for comparison with Methyl-SNP-seq for variant calling. For a fair comparison to avoid differences owing to the choice of variant calling pipeline (Cornish and Guda 2015), we processed the JIMB WGS data set using the same strategy as for the human Methyl-SNP-seq: (1) shortening the paired-end reads to 99 bp; (2) trimming Illumina adapter; (3) Bowtie 2 mapping for the paired-end reads; (4) removing multiple alignments and PCR duplicates using SAMtools (version 1.14) (Li et al. 2009) markdup; and (5) removing multiple mapping using the inhouse script (https://github.com/elitaone/Methyl-SNP-seq/tree/main/Read_Processing/MarkUniread.py). To achieve a similar coverage, we downsampled to use 1.6 billion filtered JIMB WGS reads (Read1 and Read2) for variant calling.

#### Data processing for human NA12878 whole-genome bisulfite sequencing

Whole-genome bisulfite sequencing (WGBS) of human NA12878 generated by ENCODE (ENCSR890UQO) was compared with Methyl-SNP-seq for methylation quantification. For a fair comparison, we reduced the paired-end WGBS data to 99-bp long and trimmed the Illumina adapters. Next, the read pairs were aligned to the human GRCh38 genome using Bismark (version 0.22.3). The properly paired reads were further filtered before methylation determination by: (1) removing PCR duplicates using SAMtools markdup; (2) filtering out alignments having incomplete bisulfite conversion using Bismark filter_non_conversion. The two ENCODE replicates were combined to about 1.6 billion filtered reads (Read1 and Read2) for methylation quantification.

#### Variant calling and SNV comparison

We performed variant calling on the filtered data set as mentioned above using GATK (version 4.1.8.1), following GATK best practice recommendations for germline short variant discovery. First, BaseCalibration (BaseRecalibrator and ApplyBQSR) was applied to the filtered data set to calibrate the systematic errors made by sequencing. Next, the calibrated reads were used for variant calling using HaplotypeCaller. Finally, FilterVariantTranches was applied to filter raw SNVs using ‐‐info-key CNN_1D and ‐‐snp-tranche 99 ‐‐indel-tranche 99. For human targeted Methyl-SNP-seq sequencing, an additional filter “DP < 6” was applied to remove SNPs with low coverage. In this study, only SNVs on the somatic chromosomes, Chr X and Chr M, were reported and used for analysis.

Methyl-SNP-seq genetic variants were defined as the common SNVs identified by both deconvoluted read and Read2. We used vcfeval from RTG Tools (version 3.11) (Cleary et al. 2014) to compare the SNVs identified by Methyl-SNP-seq and the benchmark JIMB WGS. We used Picard tools (version 2.26.11) CollectVariantCallingMetrics function to measure the transition and transversion ratio.

#### Methylation quantification

For both Methyl-SNP-seq and WGBS, the methylation information was extracted on the filtered reads and read pairs, respectively, using bismark_methylation_extractor (version 0.22.3) with the following parameters: ‐‐merge_non_CpG ‐‐bedGraph.

We also used the latest Nanopore sequencing data set of the human GM12878 cell line for methylation detection (https://github.com/nanopore-wgs-consortium/NA12878/blob/master/Genome.md) (Jain et al. 2018). The Nanopore reads (in total 8.7 million from 21 runs) were aligned to the human GRCh38 genome using minimap2 (version 2.17) (Li 2018). The methylation modification was detected using nanopolish (version 0.13.2) (Loman et al. 2015) call-methylation function.

The methylation level of UCSC annotated CpG islands (CGI) was defined as:

CGI methylation = number of methylated CpG Cs in the region/number of CpG Cs in the region.

Only the CGIs having coverage (number of CpG Cs in the region) above 50 were used for comparison between different methods.

#### Allele-specific methylation (ASM) determination

To discover the allele-specific methylation loci in the NA12878 genome, we used the heterozygous SNPs detected by Methyl-SNP-seq and confirmed in the JIMB NA12878 SNP VCF file (Zook et al. 2019). We split the Methyl-SNP-seq alignments into two groups based on the defined SNP: REF (reads having the reference SNP) and ALT (reads having the alternative SNP). The methylation status of CpG sites was extracted for each group using bismark_ methylation_extractor as previously mentioned. Finally, the differentially methylated regions between the REF and the ALT groups were detected using the DSS tool (version 2.38.0) (Feng et al. 2014) with the following threshold for callDML and callDMR function: delta = 0.1, p.threshold = 0.05.

#### Genome assembly of Methyl-SNP-seq of E. coli and mixed bacterial sample

Bacterial genomes were assembled using Velvet (version 1.2.10) based on 16.4 million and 36.7 million deconvoluted reads for *E. coli* and mixed samples, respectively. The following parameters were used for Velvet assembly to obtain the best result: for *E. coli*, k = 81 -fastq -short -exp_cov 13 -cov_cutoff 9 -min_ contig_lgth 500; for mixed sample, k = 75 -fastq -short -exp_cov 15 -cov_cutoff 8 -min_contig_lgth 500. The assembly quality was estimated using the QUAST web interface (Gurevich et al. 2013).

#### Determination of methyltransferase recognition site based on the deconvoluted reads

We randomly chose 2% deconvoluted reads to identify the methyltransferase recognition site in *E. coli* or mixed samples (0.28 million or 0.67 million reads, respectively). The 8-mers including 3 bp upstream of and 4 bp downstream from either a 5mC or unmethylated C were extracted for each read. The reads including more than one methylated C were excluded from this analysis (https://github.com/elitaone/Methyl-SNP-seq/tree/main/Methylation_Motif_Calling/motifExtraction.py -l 3 -r 4). The numbers of 8-mers containing either 5mC or 5mC and unmethylated cytosine were counted. We used Binomial statistics with Bonferroni Correction to determine the 8-mer sequences that have significantly higher methylation levels compared with the background. The *P*-value is calculated using the following formula:

P−value(ofeach8−mersequence)=1−binom.cdf(k,n,P0).

For each 8-mer sequence, *k* is the number of 8-mers having 5mC; n is the number of 8-mers having 5mC and unmethylated cytosine; P0 is the average methylation level. We used a custom script to perform this statistical analysis to extract the significantly enriched/methylated 8-mers (https://github.com/elitaone/Methyl-SNP-seq/blob/main/Methylation_Motif_Calling/identifyMotif.py, ‐‐alpha 0.0001 for Binomial Significance Levels, ‐‐mode average).

These significantly enriched 8-mer sequences were further clustered to create the motif logo using a hierarchical linkage method based on the difference between each pair of sequences (https://github.com/elitaone/Methyl-SNP-seq/tree/main/Methylation_Motif_Calling/clusterMotif.py). The number of clusters (‐‐number) can be decided based on the cluster heatmap. Specifically, in this study, we assigned the significantly enriched sequences into two clusters for *E. coli* and three clusters for the mixed sample (Fig. 6B).

## Data access

All raw and processed sequencing data generated in this study have been submitted to the NCBI Gene Expression Omnibus (GEO; https://www.ncbi.nlm.nih.gov/geo/) under accession number GSE206253.

## Supplementary Material

Supplemental Material
