# Supplementary material for: Methyl-SNP-seq reveals dual readouts of methylome and variome at molecule resolution while enabling target enrichment
Source: Genome Res. 2022 Nov-Dec;32(11-12):2079–91. doi: 10.1101/gr.277080.122 (PMC9808626; doi:10.1101/gr.277080.122)
Supplement: Supplemental Material [file supp_32_11-12_2079__DC1.html]

Methyl-SNP-seq reveals dual readouts of methylome and variome at molecule resolution while enabling target enrichment — Supplemental Material 

# Methyl-SNP-seq reveals dual readouts of methylome and variome at molecule resolution while enabling target enrichment

## Supplemental Material

- SupplementaryCode.tar.gz
- Supplemental\_Materials.pdf
- Supplemental\_Tables.xlsx
