# Supplementary material for: Methyl-SNP-seq reveals dual readouts of methylome and variome at molecule resolution while enabling target enrichment
Source: Genome Res. 2022 Nov-Dec;32(11-12):2079–91. doi: 10.1101/gr.277080.122 (PMC9808626; doi:10.1101/gr.277080.122)
Supplement: Supplemental Material [file supp_gr.277080.122_SupplementaryCode.tar.gz › SupplementaryCode/Read_Processing/README.pdf]

Table of Contents

Introduction 2

Adapter Trimming: TrimRead.py 3

Reference-free Read Deconvolution: DeconvolutionConversion_v2.py 4

Reference-dependent Read Deconvolution: DeconvolutionWithCalibration package 5

Read Deconvolution Correction: DeconvolutionUnmatchCorrect.py 7

Removal of multiple mapping reads: MarkUniread.py 8

Removal of duplicated reads: MarkDup.py 9

Addition of XM tag: AddXMtag.py 10

Methyl-SNP-seq README

Author: Bo Yan, yan@neb.com
Revision Date: Aug 20, 2022

Introduction

Methyl-SNP-seq takes advantage of the double-stranded nature of DNA to duplicate the sequence information into a linked copy to the original strand. The copied strand replaces the cytosine with 5mC, so it is resistant to bisulfite conversion. After bisulfite conversion, the copied strand conserves its original four nucleotide content while the original strand undergoes deamination at unmethylated cytosines. The principle of Methyl-SNP-seq is summarized in the following figure.

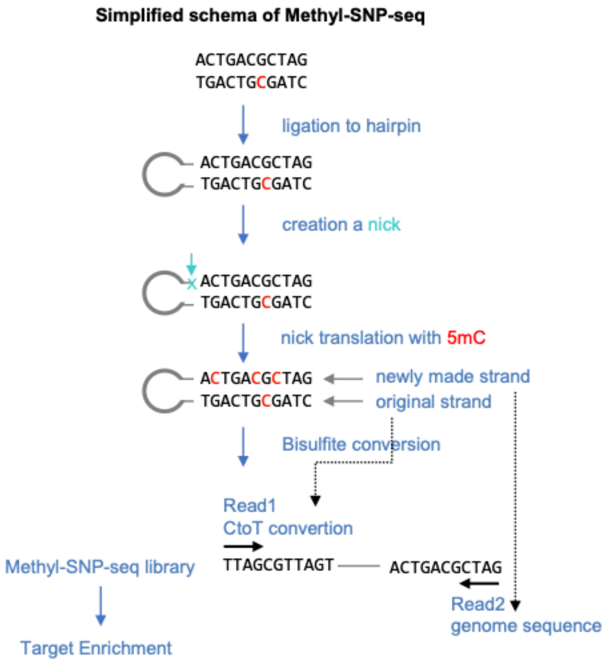

Figure: Principle of Methyl-SNP-seq

The scripts included in the ReadProcessing folder are used for processing the Methyl-SNP-seq sequencing reads, including: trimming adapters, Read Deconvolution, removal of duplicates, removal of multiple mapping, addition of XM tag for methylation extraction.

Functions

Adapter Trimming: TrimRead.py

Synopsis:
TrimRead.py generates and runs a bash script containing the commands to trim the Illumina adapter as well as the hairpin adapter sequence from the paired-end reads.
The trimming of the adapter sequence is performed using trim_galore.
The hairpin adapter sequence is provided in the Methyl-SNP-seq manuscript.
Can change the trim_galore commands using custom options.

Requirement:
Python 2.7
trim_galore (used version 0.6.4) and cupadapt is preinstalled.
Bash shell is enabled and executable in the PATH: #!/bin/bash

Usage:

```
$python TrimRead.py --Read1 TestSeq.1.fastq.gz --Read2 TestSeq.2.fastq.gz --name TestSeq
```

Parameters:

--Read1, --Read2:
Full path of the illumina sequencing read1, read2 fastq or fastq.gz file.

--name:
Name for the output fastq files, e.g. TestSeq
The resulting adapter removed Read1 and Read2 fastq files that are used for next step Read Deconvolution will be named as:
TestSeq_hairpin_R1_val_1.fq and TestSeq_hairpin_R2_val_2.fq
These files are saved under the current working directory.

--cores: Default 1
Number of cores to be used for trim_galore trimming. It is used as –cores/-j option for trim_galore.

--path_to_cutadapt, --path_to_trimgalore: Optional
Use this option to specify a path to the cutadapt or trim_galore executable,
e.g. /mnt/home/yan/exe/TrimGalore-0.6.4/trim_galore, /mnt/home/yan/exe/cutadapt
Else by default it is assumed that cutadapt or trim_galore is executable in the PATH.

Reference-free Read Deconvolution: DeconvolutionConversion_v2.py

Synopsis:
DeconvolutionConversion_v2.py performs reference-free Read Deconvolution of Methyl-SNP-seq paired-end Read1 and Read2.
This is the Deconvolution without Base Calibration, for example Methyl-SNP-seq of microbiome genomic DNA.
Read Deconvolution compares the same sequencing cycle of a paired read, including the following actions (Figure: Principle of Methyl-SNP-seq):
(1) Base determination and methylation extraction. For the same Illumina cycle, if Read1 base is a C and Read2 base is a C, it results in a C in the Deconvoluted Read and a 5mC in the methylation report; while if Read1 base is a T and Read2 base is a C, it results in a C in the Deconvoluted Read and an unmethylated C in the methylation report.
(2) Base quality score adjustment. For the mismatching positions that Read1 bases are different from Read2 bases except for the Read1-T Read2-C case, Read1 bases are used but the sequencing quality scores are adjusted to 0 in the Deconvoluted Reads.
This Deconvolution step generates a fastq file containing the Deconvoluted Reads and a methylation report containing the methylation status of each C in each Deconvolution Read.

Requirement: Python 2.7

Usage:

```
$python DeconvolutionConversion_v2.py --Read1 TestSeq_hairpin_R1_val_1.fq --Read2
TestSeq_hairpin_R2_val_2.fq --name TestSeq
```

Parameters:

--Read1, --Read2:
Illumina and hairpin adapters removed Read1 and Read2 fastq files. Only take the uncompressed fastq file.

--name:
Name for output files.
The resulting fastq file containing Deconvoluted Reads will be named as, e.g., TestSeq_DeconvolutedRead.fq.

The resulting methylation report will be named as, e.g., TestSeq.Deconvolution.5mC.
The methylation report is 0-coordination, e.g.

```
A00336:A00336:HV7F7DRXX:1:1101:10004:10927	C0C2C11C13C20C21C22C25C41C45C51C52C53C60M63C70M71C74
```

1st column is the readID
2nd column marks the methylation status of C in the deconvoluted read: C for unmethylated cytosine and M for 5mC

These files are saved under the current working directory.

Note:
The current version does not run multiple threads.

Reference-dependent Read Deconvolution: DeconvolutionWithCalibration package

Synopsis:
This package performs reference-dependent Read Deconvolution of Methyl-SNP-seq paired end Read1 and Read2.
This is the Deconvolution with Base Calibration, for example Methyl-SNP-seq of human genomic DNA.
The step (1) Base determination and methylation extraction applies the same principle as the Reference-free Read Deconvolution. But Reference-dependent Read Deconvolution applies a statistical model for the base quality score adjustment as shown below.
(2) Base quality score adjustment. For the mismatching positions, by comparing to the reference genome, a Bayesian probability is calculated, which reflects the likelihood of being able to trust the Read1 base. Therefore, Read1 bases are used but the sequencing quality scores are adjusted based on the Bayesian probability in the deconvoluted reads.

Requirement:
Python 2.7, pathos (used version 0.2.6) and pandas (used version 0.24.2) module preinstalled.
bowtie2, samtools and bedtools are preinstalled.
Bash shell is enabled and executable in the PATH: #!/bin/bash

Usage:
To perform Reference-dependent Read Deconvolution, download the DeconvolutionWithCalibration directory which contains __main__.py and src folder.
Then run the command as follows:

```
$python DeconvolutionWithCalibration --Read1 TestSeq_hairpin_R1_val_1.fq --Read2
TestSeq_hairpin_R2_val_2.fq --name TestSeq --reference hg38.fa --bowtie2_reference hg38
```

Parameters:

--Read1, --Read2:
Illumina and hairpin adapters removed Read1 and Read2 fastq files. Can take both the compressed fastq.gz or uncompressed fastq files.

--name:
Name for output files, e.g., TestSeq

- TestSeq_DeconvolutedRead.fq: fastq file containing Deconvoluted Reads
- TestSeq.Deconvolution.5mC: methylation report
- TestSeq.BaseCalibration.table: base calibration table for Bayesian modeling
- TestSeq.BaseCalibration.probability: Bayesian probability table

--reference: Required
Reference genome (.fa) used in bedtools getfasta function.

--bowtie2_reference: Optional
bowtie2 reference generated by bowtie2-build based on the reference genome. This is used for bowtie2 mapping.
If provided, skip the reference build step, else the bowtie2 reference will be built based on reference genome which may take some time.

--percent: float, Default 0.05
Subsample input reads for base calibration.

--vcf: Optional
A vcf file containing known SNP positions. If provided, the positions listed in this vcf file are ignored from base calibration.
Several variant files can be merged into one using picard.jar MergeVcfs function.

--smp: Optional, Default 1
Number of threads used for bowtie2 mapping, which is used as -p for bowtie2. Need to assign enough memory to run multiple threads.

--dir: Optional
Directory (full path) to save the output files.
If not provided, the output files are saved at current working directory.

--path_to_bowtie2, --path_to_bedtools, --path_to_samtools: Optional
Use this option to specify a path to bowtie2/bedtools/samtools executable, e.g., /usr/bin/bowtie2
Else by default it is assumed that bowtie2/bedtools/samtools is executable in the PATH.

Note:
(1) The bowtie2-build step is time consuming for human genome, so provide the premade bowtie2 reference if possible.

(2) This step generates large temporary files so make sure there is enough space.
The following gives an estimation of the size of temporary files:

```
Size of temporary files starting with an uncompressed fastq file pair having 850
million 100bp reads:
       uncompressed files: Read1.fq/Read2.fq 165G
       downsample with 0.02: TestSeq.downsample.R1.fq and TestSeq.downsample.R2.fq
       3.3G, which contains 16 million reads
       mapping sam file: TestSeq_deconvoluted_R1.sam 4.3G
       base calibration file: TestSeq_deconvoluted_R1.compareBase.txt 24G,
       TestSeq_R2.compareBase.txt 28G, TestSeq.BaseCalibration.probability
```

(3) The Deconvolution step uses pathos multiple threads to speed up. Make sure to assign enough memory to run multiple threads.
The number of threads used depends on the available number of CPU with a limit 8, which can be detected using pathos.helpers.cpu_count().
If want to use a given number of threads n, change the setting of nodes=n in the Script /~src/DeconvolutionCalibration_v2.py (Line 316): pool = ProcessPool(nodes=n).

(4) Time estimation
The following gives an estimation of the time to run each step.

```
Timing of Starting with an uncompressed fastq file pair having 850 million reads:
       Step 2 Downsample step: 45min
       Step 3 conversion of Read1: 7min
       Step 4 bowtie2 mapping with -p 4: 1h20min
       Step 5 Base Calibration in the presence of a vcf file: 2h35min
       Step 6 Deconvolution with base calibration with multiple threads 8 nodes:
       2h21min
```

Read Deconvolution Correction: DeconvolutionUnmatchCorrect.py

Synopsis:
This script is used to correct some of the unmatched read pairs that Read1 and Read2 bases are not matched properly due to insertion of deletion.
The output fastq files containing the corrected read pairs can be deconvoluted using DeconvolutionWithCalibration or DeconvolutionConversion_v2.py as explained above.

Note:
The selection of -F 4 and -F 256 will be performed by this script, so do not need to apply this selection on the Unmatched Read1 and Unmatched Read2 bam/sam files. The Unmatched bam files do not need to be sorted by coordination.

Requirement:
Python 2.7, numpy (used version 1.16.6) module preinstalled.
samtools preinstalled.
Bash shell is enabled and executable in the PATH: #!/bin/bash

Usage:

```
$python DeconvolutionUnmatchCorrect.py --Read1 TestSeqUnmatch_R1.bam --Read2
TestSeqUnmatch_R2.bam --name TestSeq
```

Parameters:

--Read1: alignment of unmatched Read1 bam/sam file
Unmatched Read1 bam/sam file:
A Read1 fastq file, which contains the reads that cannot be deconvoluted using DeconvolutionWithCalibration or DeconvolutionConversion_v2.py, is aligned using bismark mapping.
The generated bam/sam file is used as Unmatched Read1 bam/sam file.

--Read2: alignment of unmatched Read2 bam/sam file
Unmatched Read2 bam/sam file:
The Read2 fastq file, which contains the reads that cannot be matched using DeconvolutionWithCalibration or DeconvolutionConversion_v2.py, is aligned using bowtie2 mapping.
The generated bam/sam file is used as Unmatched Read1 bam/sam file.

--name:
Name for output Read1 and Read2 files having the corrected matched reads, e.g., TestSeq.
The output files TestSeq _unmatchcorrect.R1.fastq and TestSeq_unmatchcorrect.R2.fastq can be used as input files for Read Deconvolution as shown above.

--path_to_samtools: Optional
Use this option to specify a path to the samtools executable,
e.g. /usr/bin/samtools.
Else by default it is assumed that samtools and bedtools is executable from the PATH.

--dir: Optional
Directory (full path) to save the output files.
If not provided, the output files are saved at current working directory.

Removal of multiple mapping reads: MarkUniread.py

Synopsis:
MarkUniread.py removes the multiple mapping based on **bowtie2 mapping**. Only the reliable (uniquely) mapping is saved in the output sam file.
Reliable mapping is defined as:
(1) Flag != 4, 256, 2048
(2) XS tag not present or AS tag != XS tag. See bowtie2 manual for definition of XS and AS tag.
(3) If --MAPQ cutoff is provided, only mapping above the MAPQ cutoff is saved.
Other alignment tools e.g. bwa mem uses different strategy to label the multiple mapping, therefore this script only works with the bowtie2 mapping.

Requirement:
Python 2.7, numpy (used version 1.16.6) module preinstalled.

Usage:

```
$python MarkUniread.py --input TestSeq_DeconvolutedRead.sam --output
TestSeq_DeconvolutedRead.uni.sam
```

Parameters:

--input:
A sam file containing the **bowtie2 mapping** of the deconvoluted reads.

--output:
A sam file containing the bowtie2 mapping of deconvoluted reads with removal of multiple mapping. The output file keeps the order of mapping as in the input file.

--MAPQ: Default 0. Int
If this MAPQ cutoff is provided, only mapping >= the MAPQ cutoff is saved in output.

Removal of duplicated reads: MarkDup.py

Synopsis:
MarkDup.py removes the duplicated reads for Single end mapping, e.g., Deconvoluted Read mapping with bowtie2.
Here duplicated reads are defined as single end reads mapped to the same chr and locus and have the same sequence in col 10. For duplicates, save one copy with the highest MAPQ score in the output sam file.

Note:
Do not perform -F 256 or -F 1024 or -F 2048 selection in this step. Apply these filters in advance if necessary.
This script does not consider AS and XS tag, therefore should apply MarkUniread.py before this step if filtering multiple mapping is required.

Requirement: Python 2.7

Usage:

```
$python MarkDup.py --input TestSeq_DeconvolutedRead.uni.sam --output
TestSeq_DeconvolutedRead.uni.nodup.sam
```

Parameters:

--input:
A sam file sorted by coordination containing the deconvoluted reads.

--output:
A sam file sorted by coordination containing the deconvoluted reads with removal of duplicates.

Addition of XM tag: AddXMtag.py

Synopsis:
AddXMtag.py adds a XM tag to each mapping in the input sam file of the Deconvoluted Read. XM tag is defined by bismark to labeling the methylation status. Therefore, the methylation status at each position could be further extracted using bismark_methylation_extractor.
The determination of methylation status of the deconvoluted reads is like the bismark principle as shown below.

```
To report XM tag based on the deconvolution report:
    nonC: position is not a C or Methylated C

    If a position is a C (or Methylated C) in the deconvolution report, and also C on genome:
        Find the methylation context based on the seq in genome, use x,h,z,u or Capital in XM tag following bismark rules.
    If a position is nonC in either deconvolution report or genome:
        Use '.' in XM tag in the corresponding position in SEQ
    If a position is a C on genome but is nonC in deconvolution report:
        Use '.' in XM tag.
    If a position is C in deconvolution report, but nonC on genome (mismatch) or this base is a insertion (I in cigar) not present in genome:
        Use '.' in XM tag.

    The C context is determined based on the corresponding genome, in other words based on the mapping strand from 5' to 3' direction:
        For read mapping to reverse strand (Flag 16),
        e.g. Read in fastq 5'-GcGT-3', SEQ=ACgC (corresponding to top strand 5'->3' direction), bottom genome=5'-GcGT-3', top genome='5-ACgC-3',
        the context is cG depending on the bottom genome .z.., so the XM tag corresponding to SEQ in bam file is reverse of '.z..' -> '..z.'

    So for C at mismatching/SNP site or before mismatching/SNP site, the C context calling could be not accurate.

There is Difference between AddXMtag.py and bismark for calling C coming in front of a deletion (^):
AddXMtag.py decides the context based on the genome context:
        col10:      TTTC^^AAATTATTTTGTGATGTGTGTGTTTAATTTATAGAGTTTAATTTTTTTTTTTTATAGGGTAGTTTGGAAATAT
        my XM:      .h.X  ...h..h.............z...h..h.h.x.........hh...h....h......x..........h.h
    top genome:   attctcagaaactactttgtgatgtgtgcgttcaactcacagagtttaacctttcttttcatagggcagtttggaaacactc
bismark labels the C in front of deletion depending on the context of sequence (CAA -> H):
        col10:      TTTC^^AAATTATTTTGTGATGTGTGTGTTTAATTTATAGAGTTTAATTTTTTTTTTTTATAGGGTAGTTTGGAAATAT
    bismark XM:     .h.H  ...h..h.............z...h..h.h.x.........hh...h....h......x..........h.h
    top genome:   attctcagaaactactttgtgatgtgtgcgttcaactcacagagtttaacctttcttttcatagggcagtttggaaacactc

No Difference between AddXMtag.py and bismark for calling C coming in front of an insertion (-):
AddXMtag.py decides the context based on the genome context:
        col10:      TTTC-TTTTTTTTGGGCTTTAGTTTCTTTTTTGGTAAAACGGGGATGGTAATGGGATATTCTTAGGGGGTGTATGA
        my XM:      ...H.....hhx....Hh.x.....Hh.h..........Z................h.hhH.x........h....
    top genome:   gatttc atttccctgggcctcagtttcctctttggtaaaacggggatggtaatgggacaccctcagggggtgcatgagg
bismark labels the C in front of insertion based on the genome context (CAT -> H):
        col10:      TTTC-TTTTTTTTGGGCTTTAGTTTCTTTTTTGGTAAAACGGGGATGGTAATGGGATATTCTTAGGGGGTGTATGA
    bismark XM:     ...H.....hhx....Hh.x.....Hh.h..........Z................h.hhH.x........h....
    top genome:   gatttc atttccctgggcctcagtttcctctttggtaaaacggggatggtaatgggacaccctcagggggtgcatgagg

No Difference between AddXMtag.py and bismark for calling C at or coming in front of mismatch.

Bismark tags needed for bismark extractor:
    XM-tag (methylation call string)
    XR-tag (read conversion state for the alignment)
    XG-tag (genome conversion state for the alignment)
```

Requirement:
Python 2.7, pathos module (used version 0.2.6) preinstalled.
bedtools and samtools preinstalled.
Bash shell is enabled and executable in the PATH: #!/bin/bash

Usage:

```
$ python AddXMtag.py --input TestSeq_DeconvolutedRead.uni.nodup.sam  --report
TestSeq.Deconvolution.5mC --name TestSeq --output_dir /Users/tmp --reference hg38.fa --
thread
```

Parameters:

--input:
A sam file that is generated by aligning the Deconvoluted Read to the reference genome.
For Methly-SNP-seq data analysis, need to remove the multiple mapping and PCR duplicates before this step. So the output of MarkDup.py can be used as input sam file.

--report:
A methylation report generated during Deconvolution Step.

--name:
Name for output files, e.g. TestSeq
TestSeq.XMtag.sam: a sam file saving all the alignments with XMtag added.
TestSeq.noXMtag.sam: a sam file saving all the alignments that cannot be added XMtag: unmapped reads, reads without information in deconvolution report or reads that cannot be analyzed for methylation context (at the boundary of chr).
The reads in output sam files keep the same order as in the input sam file.

--reference:
Reference genome (.fa) used to align the Deconvoluted Reads.

--dir: Optional
Dir for saving output files, not required.
If not provided, output files are saved in the current working dir.

--thread: Optional
Add --thread as shown in the Usage to run multiple threads using pathos if input Read1.sam is a large file.
The sam file will be split into subfile containing 30 million mapping. So in the current version the input sam file should NOT have more than 2970 million mapping with --thread option.
Need to assign enough space and memory with this option. See following note (5) for more details.

--path_to_samtools, --path_to_bedtools: Optional
Use this option to specify a path to the samtools/bedtools executable,
e.g. /usr/bin/samtools or /usr/bin/bedtools.
Else by default it is assumed that samtools/bedtools is executable from the PATH.

Note:
(1) The suspending part in SEQ (S in cigar) is not shown in XM tag, so len(XM) = sum of cigar M/I.

(2) Do not consider the alignment that has hard clip in Cigar (H).

(3) Deconvolution report may contain entries without methylation information (col2 is empty). In this case, report XM tag using '.' for all the positions.

(4) AddXMtag.py performs -F 4 to remove the unmapped reads, but does not apply other Flag tag filtering.

(5) Add --thread to run multiple threads (using pathos) to speed up for a large input file.

With --thread option, the input sam file is split into sub sam files having 30 million alignments each. Current version allows maximal 99 sub files. So, the maximum number of alignments in the input sam file is 2970 million (2970=99*30). If the input has more alignments than this number, either splitting the input sam to run separately or increasing the number (split -l number) of mappings assigned in each sub file in the Script Line 714.
The size of a sub sam file having 30 million alignments is 8.4G for 100bp Read1. Each sub sam file requires about 10-15G memory. Make sure to assign enough memory and space to run multiple threads.
The number of threads used depends on the available number of CPU with a limit 8, which can be detected using pathos.helpers.cpu_count(). If want to use a given number of threads n, change the setting of nodes=n in the Script Line 732: pool = ProcessPool(nodes=n).

(6) Time estimation
It needs about 5h to finish for an input sam file having 470 million alignments with --thread option with 8 threads.
