# Supplementary material for: Methyl-SNP-seq reveals dual readouts of methylome and variome at molecule resolution while enabling target enrichment
Source: Genome Res. 2022 Nov-Dec;32(11-12):2079–91. doi: 10.1101/gr.277080.122 (PMC9808626; doi:10.1101/gr.277080.122)
Supplement: Supplemental Material [file supp_gr.277080.122_Supplemental_Materials.pdf]

Methyl-SNP-seq reveals dual readouts of methylome and variome at molecule resolution while enabling target enrichment

Bo Yan, Duan Wang, Romualdas Vaisvila, Zhiyi Sun and Laurence Ettwiller

Supplemental Material

Supplemental Figures

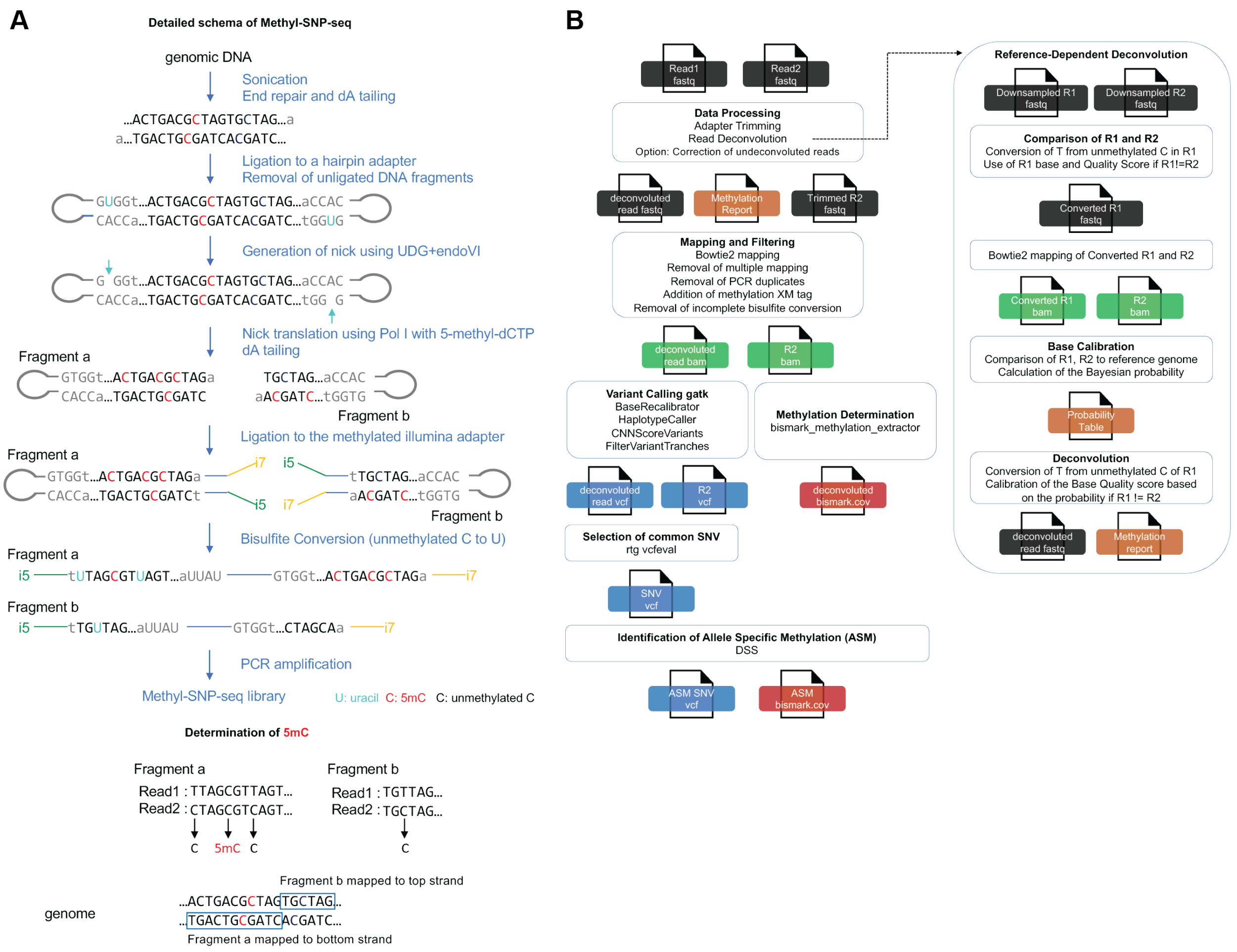

Supplemental Figure 1:
(A) Detailed description of the Methyl-SNP-seq experimental workflow. (B) Flowchart illustration of the analysis of Human Methyl-SNP-seq data. R1 and R2 stand for Read1 and Read2.

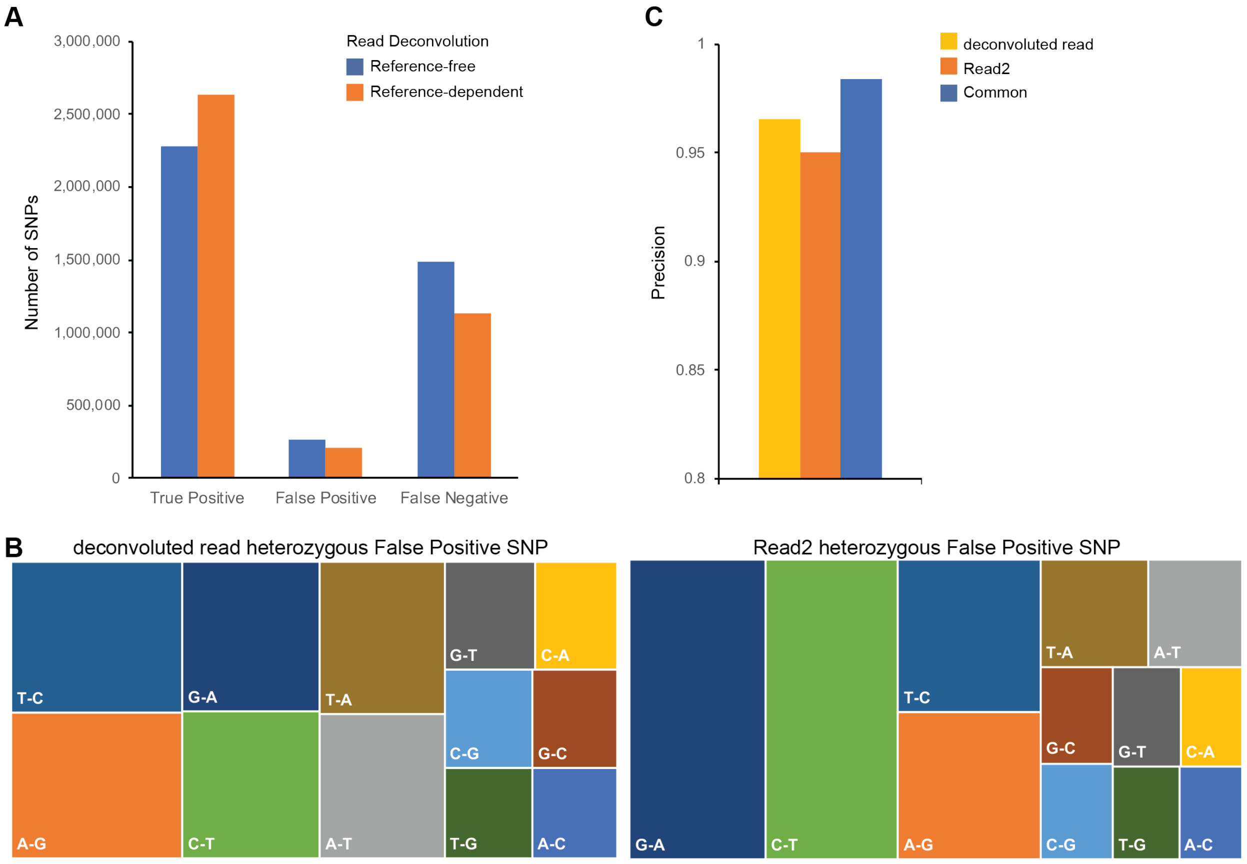

Supplemental Figure 2: Comparison of SNP calling with different strategies.
The defined SNPs were benchmarked against JIMB WGS data. (A) Methyl-SNP-seq Replicate 1 data was used for SNP calling using: Reference-free deconvoluted read and Reference-dependent deconvoluted read. (B) Characterization of the false positive heterozygous SNPs defined by deconvoluted read or Read2. (i.e. T-C means in the VCF file REF=T while ALT=C). (C) Precision of SNPs defined using deconvoluted read and Read2. The common SNPs are those detected by both deconvoluted read and Read2.

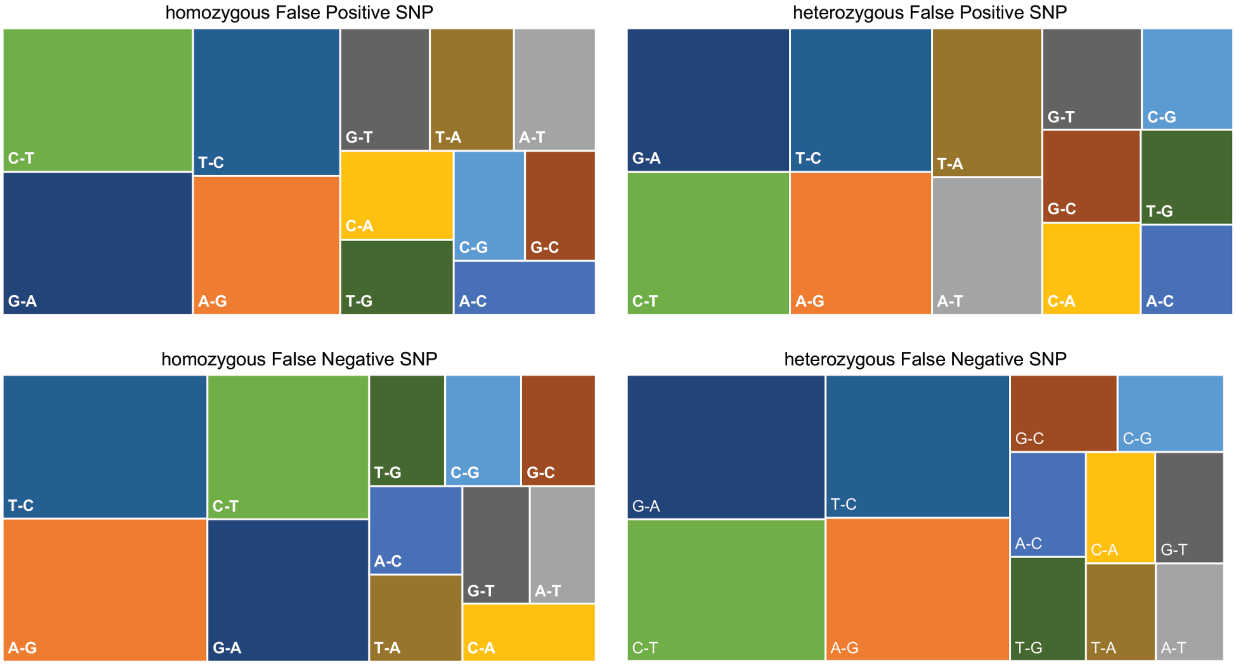

Supplemental Figure 3: Characterization of the Methyl-SNP-seq defined false positive or negative SNPs. The Methyl-SNP-seq defined SNPs were benchmarked against JIMB WGS defined SNPs. (i.e. T-C means in the VCF file REF=T while ALT=C).

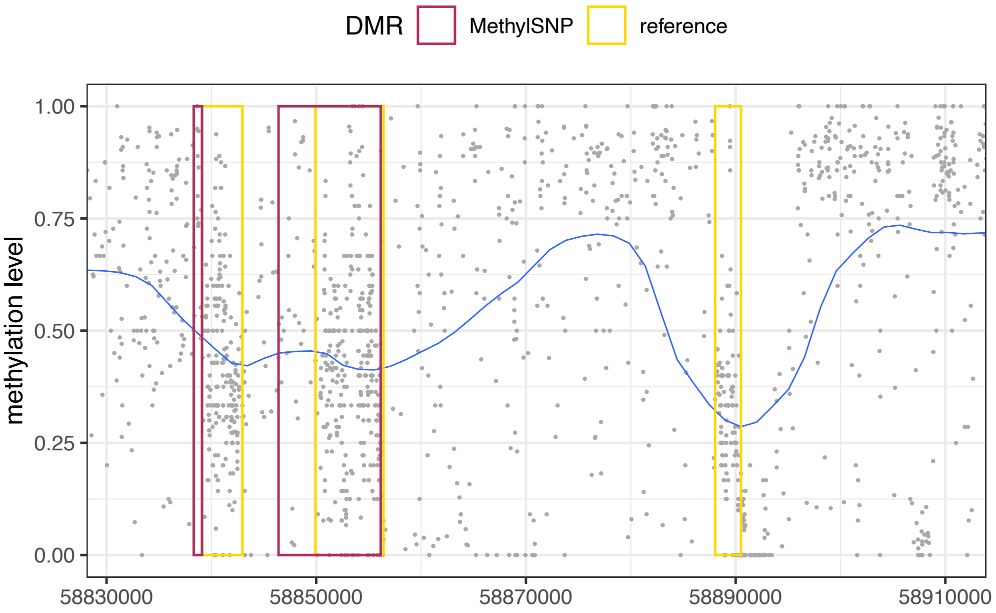

Supplemental Figure 4: CpG methylation profile and DMRs near the imprinted *GNAS* gene. Coordinates on the x axis correspond to positions on Chr 20 (GRCh38). Red box: Methyl-SNP-seq detected DMRs (the right 2 DMRs in Figure 4B examples). Yellow box: reported DMRs.

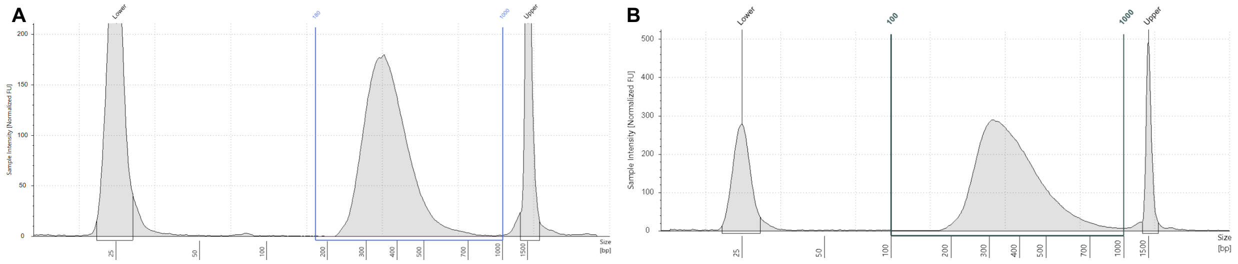

Region Table

From [bp]	To [bp]	Average Size [bp]	Conc. [pg/µl]	Region Molarity [pmol/l]	% of Total	Region Comment	Color
180	1000	376	663	2840	91.74		

Region Table

From [bp]	Average Size [bp]	Conc. [pg/µl]	Region Molarity [pmol/l]	% of Total	Region Comment	Color
100	384	1600	6960	96.00		

Supplemental Figure 5: Library size distribution measured by the high sensitivity DNA screen tape (Agilent).
(A) Methyl-SNP-seq sequencing library with 250 bp sonication protocol. (B) NEB Ultra II sequencing library with 250 bp sonication protocol.

Supplemental Text

Library size distribution

In theory, at the end of the first ligation and after USER treatment, a double stranded DNA molecule flanked by both loop adaptors should have two nicking sites in opposite ends (**Supplemental Figure 1A**). If the nick translation on both nicking sites is starting at the same time, the initial fragment breaks roughly in the middle - this reduces the size of the fragment by half. Nonetheless after deamination, the linked strands opened through the loss of complementarity, doubling the size. Thus, in theory, the final library should be of the same size as the sonicated genomic DNA (plus the size of the adapters). In practice the Methyl-SNP-seq library is slightly shorter (average size 376 bp, **Supplemental Figure 5A**) than the size of the corresponding standard genomic DNA sequencing library obtained using the same 250 bp sonication protocol (average size 384 bp, **Supplemental Figure 5B**). This result indicates that Methyl-SNP-seq follows closely the scenario described above. Importantly, the Methyl-SNP-seq library inserts sizes results in a tight distribution with most of the fragments measuring between 200 and 500 bp.

Supplemental Tables

Supplemental Table 1: Picard AlignmentSummaryMetrics.

Supplemental Table 2: Picard VariantCallingMetrics.

Supplemental Table 3: Picard GCBiasMetrics.

Supplemental Table 4: Methyl-SNP-seq reported allele-specific DMRs overlap with known imprinted clusters and associated ASDMRs.
Methyl-SNP-seq reported allele-specific DMRs which overlap or near (within the window of upstream 10 kb to downstream 10 kb) the known allele specific DMRs (Fang et al. 2012) are listed in this table.

Supplemental Table 5: Association of ASDMRs with different genomic features.

Genomic regions	Biological Significance	Assay type and data source/reference	overlapping with ASDMR	overlapping with random CpG regions	Fold change
open chromatin	open chromatin	ATA-seq (ENCSR637XSC)	3448	3650	0.9
H3K27ac	active enhancer	ChIP-seq (ENCSR000AKC (Creyghton et al. 2010))	1529	1773	0.9
H3K4me1	enriched at active and primed enhancers	ChIP-seq (ENCSR000AKF (Rada-Iglesias 2018))	2397	1887	1.3
H3K4me1 - H3K4me3	general enhancers including active and primed enhancers, non-promoters	(Creyghton et al. 2010)	1594	1090	1.5
H3K4me3	promoters and active transcription	ChIP-seq (ENCSR057BWO (Mikkelsen et al. 2007))	1142	1716	0.7
H3K9me3	heterochromatin; frequently coexist with methylation	ChIP-seq (ENCSR000AOX)	513	355	1.4

Supplemental Table 6: Picard target enrichment HsMetrics for targeted Methyl-SNP-seq.

Supplemental Protocol

Buffer used:
LowTE buffer: 1 mM Tris pH8.0 and 0.1 mM EDTA

Preparation of 20 µM annealed hairpin adapter:
10X annealing buffer: 100 mM Tris pH8.0 and 1M NaCl
Dilute the hairpin adapter to 20 µM in 1X annealing buffer. Anneal as follows: incubate at 80 °C for 2 min then ramp temperature to 25 °C at a rate of 0.1 °C per second. The annealed hairpin adapter is stored at -20 °C.
The sequence of the custom hairpin adapter (46 bp) is:
5'-(p)CCACGACGACGACGACGAGCGTTAGGCTCGTCGTCGTCGTCGUGGT-3'

Preparation of 50 µl 10 mM d(A,T,G)TP mix:

100 mM dATP	5 µl
100 mM dGTP	5 µl
100 mM dTTP	5 µl
H2O	35 µl
Total	50 µl

1. Fragmentation of genomic DNA (gDNA)

Sonicate 2 µg NA12878 gDNA (with addition of 1 ng non-methylated lambda gDNA as spike-in) in 50 µl 10 mM Tris pH8.0 using Covaris S2 with the following setup: 250 bp protocol, Duty Cycle: 10%, Intensity: 5, Cycles/burst 200, Time 80s.

Set up two sonication (total 4 µg gDNA) for the human whole genome Methyl-SNP-seq sequencing.

2. End Repair and dA tailing using Ultra II End Repair/dA tailing module (NEB E7546)

Fragmented gDNA	100 µl
NEBNext ultra II End Prep Enzyme Mix	9 µl
NEBNext ultra II End Prep Reaction Buffer	21 µl
H2O	50 µl
Total	180 µl

Incubate at 20 °C for 30 min, then at 65 °C for 30 min, then cool down to 12 °C.

Clean up the 180 µl reaction using Oligo Clean & Concentrator (Zymo Research, D4060) with the 80 nt protocol. Elute the column with 26 µl LowTE buffer.

3. Ligation to hairpin adapter and removal of unligated DNA

dA tailed DNA from step 2	26 µl
20 µM annealed hairpin adapter	4 µl
2X Ligation Mix (NEB M0367)	30 µl
Total	60 µl

Incubate at 20 °C for 1 h.

Clean up the 60 µl reaction using 0.9X (55 µl) SPRI/Ampure beads, elute with 40 µl LowTE buffer.

ExoIII and ExoVII treatment:

Ligated DNA	40 µl
ExoIII (100U/µl) (NEB M0206)	2.5 µl
ExoVII (10U/µl) (NEB M0379)	2.5 µl
10X NEB Cutsmart buffer	5 µl
Total	50 µl

Incubate at 37 °C for 1 h.

Clean up the 50 µl reaction using Oligo Clean & Concentrator (Zymo Research, D4060) with the 80 nt protocol. Elute the column with 40 µl LowTE buffer.

Stop point: save product at -20 °C.

4. Nick translation and dA tailing

Ligated DNA from step 3	39 µl
10X NEB Buffer 2	5 µl
UDG (5U/µl) (NEB M0280)	2 µl
Endo IV (10U/µl) (M0304)	2 µl
Total	48 µl

Incubate at 37 °C for 15 min.

Then add directly to the reaction without purification:

10 mM d(A,T,G)TP mix	0.5 µl
10 mM 5-methyl-dCTP (NEB N0356)	0.5 µl
NEB DNA pol I (10U/µl) (NEB M0209)	1 µl
Total	50 µl

Incubate at 20 °C for 30 min.

To add dA tail, add directly to the reaction without purification: 1 µl 100 mM dATP (final concentration 2 mM), 2 µl Taq Polymerase (NEB M0267), incubate at 65 °C for 30 min, then cool down to 12 °C.

Clean up the 50 µl reaction using Oligo Clean & Concentrator (Zymo Research, D4060) with the 80 nt protocol. Elute the column with 14 µl 10mM Tris pH8.0.

5. Ligation to the methylated illlumina adapter

dA tailed 5mC incorporated DNA from step 4	14 µl
7.5 µM NEB methylated adapter	1 µl
2X Ligation Mix (NEB M0367)	15 µl
Total	30 µl

Note: Dilute the 15 µM NEB methylated adapter (NEB E7165 included in the E7140 kit) using 10 mM Tris pH8.0 or NEB adapter dilution buffer.

Incubate at 20 °C for 1 h.

Add volume to 50 µl and clean up using 0.8X (40 µl) SPRI/Ampure beads. Elute with 20 µl LowTE buffer.

6. Sodium bisulfite treatment

Perform the sodium bisulfite conversion using Fast Bisulfite Conversion kit (Abcam ab11727) following the manufacturer's instruction with the following modifications:
Evaporate the 20 µl DNA from step 5 into 5 µl to mix with the bisulfite reagent.
Incubate the bisulfite conversion reaction at 95 °C for 25 min.

7. PCR amplification

Perform PCR amplification using NEBNext Q5U Master Mix (NEB M0597) following the manufacturer's instruction.
In our study, we used 8 PCR cycles which produced about 50 µl 3-4 nM amplified library (**Supplemental Figure 2A**).

We used these libraries for two illumina NovaSeq runs (GSE206253):
GSM6248710: Methyl-SNP-seq_NA12878_Rep1_lane1
GSM6248711: Methyl-SNP-seq_NA12878_Rep1_lane2
GSM6248712: Methyl-SNP-seq_NA12878_Rep2_lane1
GSM6248713: Methyl-SNP-seq_NA12878_Rep2_lane2

Commands used for data analysis (Supplemental Figure 2B)

The custom scripts used for data analysis are provided in the supplemental code and on GitHub (https://github.com/elitaone/Methyl-SNP-seq/tree/main/Read_Processing/).
See details in the manual (README.pdf).

The commands shown below are used for data processing for NA12878 Methyl-SNP-seq Rep1.
The human NA12878 Methyl-SNP-seq Rep1 were sequenced in two NovaSeq runs: lane1 (SRR19719032) and lane2 (SRR19719031).
SRR19719032 Read1 and Read2 files are named as:
`Methyl-SNP-seq_NA12878_Rep1_lane1.1.fastq.gz`
`Methyl-SNP-seq_NA12878_Rep1_lane1.2.fastq.gz`

Data Processing

Command used for adapter trimming

```
python ~/Read_Processing/TrimRead.py \
--Read1 Methyl-SNP-seq_NA12878_Rep1_lane1.1.fastq.gz \
--Read2 Methyl-SNP-seq_NA12878_Rep1_lane1.2.fastq.gz \
--name Methyl-SNP-seq_NA12878_Rep1_lane1 \
--path_to_cutadapt /usr/bin/cutadapt \
--path_to_trimgalore /usr/bin/TrimGalore-0.6.4/trim_galore --core 2
```

The output files with illumina adapter and hairpin adapter removed are:
`Methyl-SNP-seq_NA12878_Rep1_lane1_hairpin_R1_val_1.fq`
`Methyl-SNP-seq_NA12878_Rep1_lane1_hairpin_R2_val_2.fq`

Command used to build Bowtie2 index (used as --bowtie2_reference in the next step)

```
bowtie2-build GCA_000001405.15_GRCh38_no_alt_analysis_set.fna
GCA_000001405.15_GRCh38_no_alt_analysis_set
```

Command used for Reference-dependent Read Deconvolution

```
python ~/Read_Processing/DeconvolutionWithCalibration
--Read1 Methyl-SNP-seq_NA12878_Rep1_lane1_hairpin_R1_val_1.fq \
--Read2 Methyl-SNP-seq_NA12878_Rep1_lane1_hairpin_R2_val_2.fq \
--name Methyl-SNP-seq_NA12878_Rep1_lane1 \
--bowtie2_reference GCA_000001405.15_GRCh38_no_alt_analysis_set \
--reference GCA_000001405.15_GRCh38_no_alt_analysis_set.fna \
--smp 4 --percent 0.05 \
--path_to_bowtie2 /usr/bin/bowtie2 --path_to_samtools /usr/bin/samtools \
--path_to_bedtools /usr/bin/bedtools
```

The output files are:
`Methyl-SNP-seq_NA12878_Rep1_lane1.BaseCalibration.probability`
`Methyl-SNP-seq_NA12878_Rep1_lane1.BaseCalibration.table`
`Methyl-SNP-seq_NA12878_Rep1_lane1.DeconvolutedRead.fq (deconvoluted reads)`
`Methyl-SNP-seq_NA12878_Rep1_lane1.Deconvolution.5mC (methylation report)`

Bowtie2 mapping

Command used for Bowtie2 mapping of deconvoluted reads:

```
bowtie2 -p 4 -x GCA_000001405.15_GRCh38_no_alt_analysis_set \
-U Methyl-SNP-seq_NA12878_Rep1_lane1.DeconvolutedRead.fq \
--rg-id A00336.HNKCMDRXX.1 --rg LB:Rep1 --rg PL:ILLUMINA \
--rg PU:HNKCMDRXX.1.ATTACTCGCCTATCCT --rg SM:NA12878 \
-S Methyl-SNP-seq_NA12878_Rep1_lane1.DeconvolutedRead.sam
```

Information for RG header required by GATK base calibration is provided using --rg and –rg-id.
Run id: A00336; flowcell id: HNKCMDRXX; flowcell lane: 1; barcode ATTACTCGCCTATCCT

The trimming and deconvolution are also performed for Rep1 lane2 as shown above.

The mapping files for Rep1 lane1 and lane2 deconvoluted reads are:
`Methyl-SNP-seq_NA12878_Rep1_lane1.DeconvolutedRead.sam`
`Methyl-SNP-seq_NA12878_Rep1_lane2.DeconvolutedRead.sam`

Commands used to merge the Rep1 lane1 and lane2 deconvoluted reads mapping and sort

```
samtools merge Methyl-SNP-seq_NA12878_Rep1_lane1.DeconvolutedRead.sam \
Methyl-SNP-seq_NA12878_Rep1_lane2.DeconvolutedRead.sam \
Methyl-SNP-seq_NA12878_Rep1.DeconvolutedRead.sam
```

```
samtools sort -o Methyl-SNP-seq_NA12878_Rep1.DeconvolutedRead.sort.sam \
Methyl-SNP-seq_NA12878_Rep1.DeconvolutedRead.sam
```

The output file is a sorted SAM file:
`Methyl-SNP-seq_NA12878_Rep1.DeconvolutedRead.sam`

Alignment filtering and addition of methylation XMtag

Command used to remove the multiple mapping

```
python ~/Read_Processing/MarkUniread.py \
--input Methyl-SNP-seq_NA12878_Rep1.DeconvolutedRead.sam \
--output Methyl-SNP-seq_NA12878_Rep1.DeconvolutedRead.uni.sam
```

The output file is a sorted SAM file:
`Methyl-SNP-seq_NA12878_Rep1.DeconvolutedRead.uni.sam`

Command used to remove the PCR duplicates

```
python ~/Read_Processing/MarkDup.py \
--input Methyl-SNP-seq_NA12878_Rep1.DeconvolutedRead.uni.sam \
--output Methyl-SNP-seq_NA12878_Rep1.DeconvolutedRead.uni.nodup.sam
```

The output file is a sorted SAM file:
`Methyl-SNP-seq_NA12878_Rep1.DeconvolutedRead.uni.nodup.sam`

Command used to combine the Rep1 lane1 and lane2 methylation reports

```
cat Methyl-SNP-seq_NA12878_Rep1_lane1.Deconvolution.5mC \
Methyl-SNP-seq_NA12878_Rep1_lane1.Deconvolution.5mC \
Methyl-SNP-seq_NA12878_Rep1.Deconvolution.5mC
```

The output file is:
`Methyl-SNP-seq_NA12878_Rep1.Deconvolution.5mC`

Command used to add XMtag

```
python ~/Read_Processing/AddXMtag.py \
--input Methyl-SNP-seq_NA12878_Rep1.DeconvolutedRead.uni.nodup.sam \
--report Methyl-SNP-seq_NA12878_Rep1.Deconvolution.5mC \
--name Methyl-SNP-seq_NA12878_Rep1 \
--reference GCA_000001405.15_GRCh38_no_alt_analysis_set.fna \
--thread --smp 8 \
--path_to_samtools /usr/bin/samtools --path_to_bedtools /usr/bin/bedtools
```

The output files are:
`Methyl-SNP-seq_NA12878_Rep1.XMtag.sam (sorted mapping with XMtag added, used as input file for bismark filter_non_conversion and/or bismark_methylation_extractor)`
`Methyl-SNP-seq_NA12878_Rep1.noXMtag.sam (sorted mapping ignored from XMtag addition)`

Supplemental References

Creyghton MP, Cheng AW, Welstead GG, Kooistra T, Carey BW, Steine EJ, Hanna J, Lodato MA, Frampton GM, Sharp PA et al. 2010. Histone H3K27ac separates active from poised enhancers and predicts developmental state. *Proceedings of the National Academy of Sciences of the United States of America* **107**: 21931-21936.

Fang F, Hodges E, Molaro A, Dean M, Hannon GJ, Smith AD. 2012. Genomic landscape of human allele-specific DNA methylation. *Proceedings of the National Academy of Sciences of the United States of America* **109**: 7332-7337.

Mikkelsen TS, Ku M, Jaffe DB, Issac B, Lieberman E, Giannoukos G, Alvarez P, Brockman W, Kim TK, Koche RP et al. 2007. Genome-wide maps of chromatin state in pluripotent and lineage-committed cells. *Nature* **448**: 553-560.

Rada-Iglesias A. 2018. Is H3K4me1 at enhancers correlative or causative? *Nature genetics* **50**: 4-5.
