# Supplementary material for: Methyl-SNP-seq reveals dual readouts of methylome and variome at molecule resolution while enabling target enrichment
Source: Genome Res. 2022 Nov-Dec;32(11-12):2079–91. doi: 10.1101/gr.277080.122 (PMC9808626; doi:10.1101/gr.277080.122)
Supplement: Supplemental Material [file supp_gr.277080.122_SupplementaryCode.tar.gz › SupplementaryCode/Methylation_Motif_Calling/TestData.zip!/TestData/Ecoli_motif_logo.pdf]

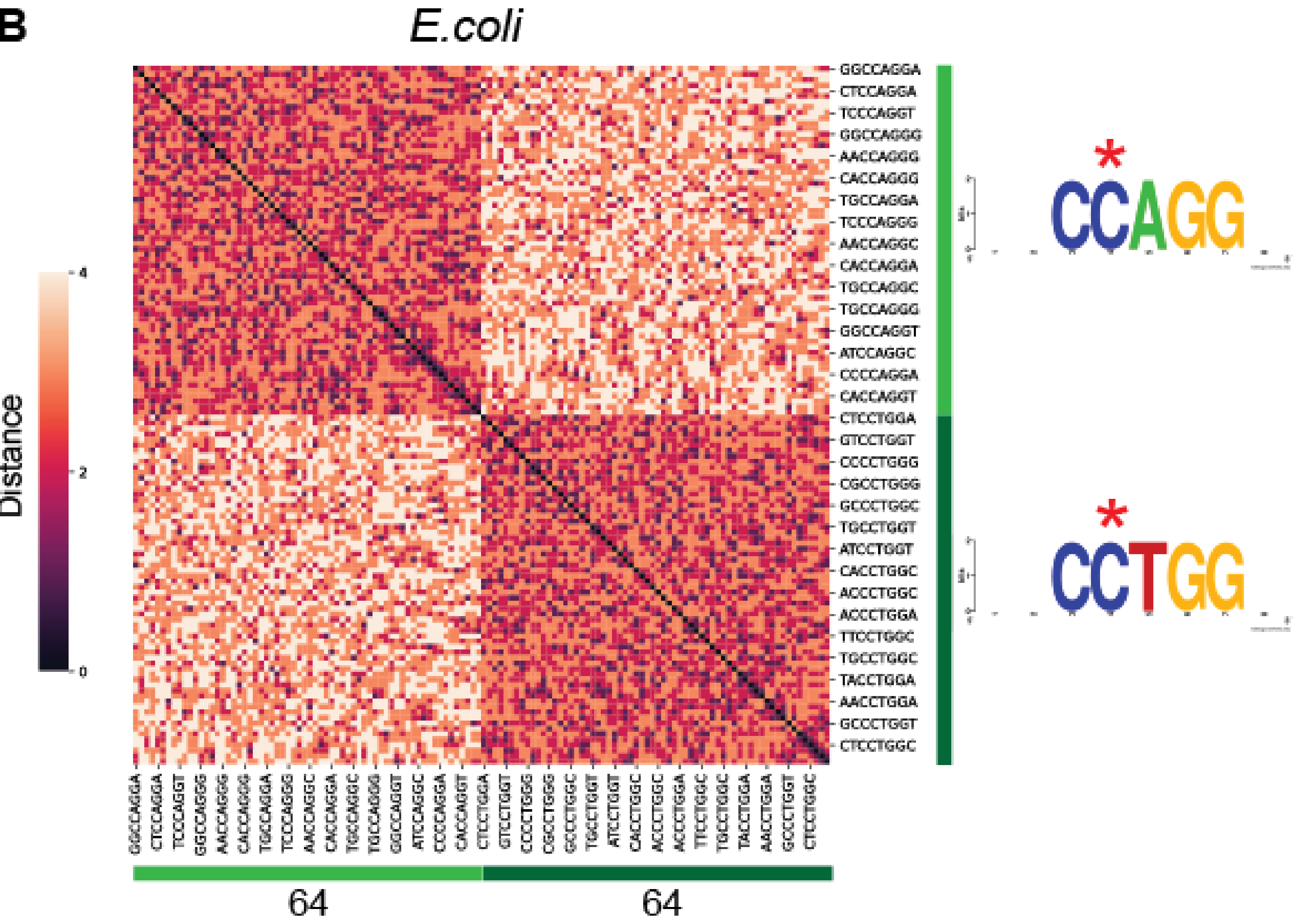

B
E.coli
Distance
4
2
0
GGCCAGGA
CTCCAGGA
TCCCAGGT
GGCCAGGG
AACCAGGG
CACCAGGG
TGCCAGGA
TCCCAGGG
AACCAGGC
CACCAGGA
TGCCAGGC
TGCCAGGG
GGCCAGGT
ATCCAGGC
CCCCAGGA
CACCAGGT
CTCCTGGA
GTCCTGGT
CCCCTGGG
CGCCTGGG
GCCCTGGC
TGCCTGGT
ATCCTGGT
CACCTGGC
ACCCTGGC
ACCCTGGA
TTCCTGGC
TGCCTGGC
TACCTGGA
AACCTGGA
GCCCTGGT
CTCCTGGC
64
64
CCAGG
CCTGG
